# *FOXE3* contributes to Peters anomaly through transcriptional regulation of an autophagy-associated protein termed DNAJB1

**DOI:** 10.1038/ncomms10953

**Published:** 2016-03-24

**Authors:** Shahid Y. Khan, Shivakumar Vasanth, Firoz Kabir, John D. Gottsch, Arif O. Khan, Raghothama Chaerkady, Mei-Chong W. Lee, Carmen C. Leitch, Zhiwei Ma, Julie Laux, Rafael Villasmil, Shaheen N. Khan, Sheikh Riazuddin, Javed Akram, Robert N. Cole, C. Conover Talbot, Nader Pourmand, Norann A. Zaghloul, J. Fielding Hejtmancik, S. Amer Riazuddin

**Affiliations:** 1 The Wilmer Eye Institute, Johns Hopkins University School of Medicine, 600 North Wolfe Street, Baltimore, Maryland 21287, USA; 2 King Khaled Eye Specialist Hospital, Riyadh 11462, Saudi Arabia; 3 Department of Biological Chemistry, Johns Hopkins University School of Medicine, 733 North Broadway, Baltimore, Maryland 21205, USA; 4 Department of Biomolecular Engineering, University of California, Santa Cruz, California 94305, USA; 5 Division of Endocrinology, Diabetes, and Nutrition, Department of Medicine, University of Maryland School of Medicine, 660 West Redwood Street, Baltimore, Maryland 21201, USA; 6 Ophthalmic Genetics and Visual Function Branch, National Eye Institute, National Institutes of Health, Bethesda, Maryland 20892, USA; 7 Laboratory of Immunology, National Eye Institute, National Institutes of Health, Bethesda, Maryland 20892, USA; 8 National Centre of Excellence in Molecular Biology, University of the Punjab, Lahore 53700, Pakistan; 9 Allama Iqbal Medical College, University of Health Sciences, Lahore 54550, Pakistan; 10 National Centre for Genetic Diseases, Shaheed Zulfiqar Ali Bhutto Medical University, Islamabad 44000, Pakistan; 11 Institute for Basic Biomedical Sciences, Johns Hopkins University School of Medicine, 733 North Broadway, Baltimore, Maryland 21205, USA

## Abstract

*FOXE3* is a lens-specific transcription factor that has been associated with anterior segment ocular dysgenesis. To determine the transcriptional target(s) of *FOXE3* that are indispensable for the anterior segment development, we examined the transcriptome and the proteome of cells expressing truncated FOXE3 responsible for Peters anomaly identified through linkage-coupled next-generation whole-exome sequencing. We found that DNAJB1, an autophagy-associated protein, was the only candidate exhibiting differential expression in both screens. We confirmed the candidacy of *DNAJB1* through chromatin immunoprecipitation and luciferase assays while knockdown of *DNAJB1* in human lens epithelial cells resulted in a mitotic arrest. Subsequently, we targeted *dnajb1a* in zebrafish through injection of a splice-blocking morpholino. The *dnajb1a* morphants exhibited underdeveloped cataractous lenses with persistent apoptotic nuclei. In conclusion, here we report DNAJB1 is a transcriptional target of FOXE3 in a novel pathway that is crucial for the development of the anterior segment of the eye.

## Abstract (web summary)

Peter's Anomaly is a developmental disorder of the eye and has been linked to mutations in a range of genes, including the transcription factor FOXE3. Here the authors use next-generation RNA sequencing and mass spectrometry to identify an autophagy-associated protein, DNAJB1 as the transcriptional target of FOXE3.

## Graphical abstract

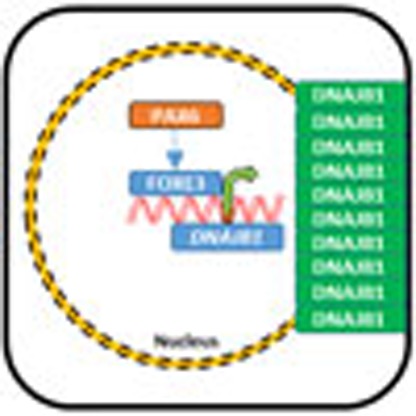

Peters anomaly (PA) is a severe developmental disorder that involves multiple tissues of the anterior segment of the eye including the iris, cornea and lens. The clinical presentation of PA includes a spectrum of central posterior corneal abnormalities with variable adhesion to the iris and/or lens^1^,^2^,^3^,^4^. In addition, Schlemm's canal and trabecular meshwork drainage structures may also be compromised, resulting in an increased risk of early-onset glaucoma^5^,^6^,^7^,^8^.

Mutations in *FOXE3, PAX6*, *PITX2*, *FOXC1*, *PITX3* and *CYP1B1* have been shown to cause isolated and syndromic PA^9^,^10^,^11^,^12^. *FOXE3* encodes a DNA-binding transcription factor that displays lens-specific expression during early development of the eye, coinciding with the formation of the lens placode^13^,^14^. Recently, Islam *et al*.^15^ demonstrated altered and loss-of-function transcriptional activity for dominant and recessive *FOXE3* mutant alleles, respectively.

In the present study, we investigated the functional consequences of a *FOXE3* non-sense allele responsible for PA in a large consanguineous pedigree. We employed next-generation sequencing-based transcriptome and mass spectrometry-based proteome profiling to determine downstream targets of mutant FOXE3. These analyses identified DNAJB1, an autophagy-associated heat shock protein (HSP) abundantly expressed in the mouse lens and human lens epithelial (HLE) cells, as the sole candidate differentially expressed in both screens. Consistent with this, morpholino-based knockdown of *dnajb1a* in zebrafish resulted in reduced eye size with cataractous lenses, mimicking symptoms of PA.

## Results

### Ascertainment and clinical evaluation of PKCC139

In an ongoing effort to investigate the genetic determinants of anterior segment dysgenesis, we recruited a large inbred family, PKCC139 (Supplementary Fig. 1a). A detailed medical and physical assessment including a slit-lamp examination was performed on enrolment to fully characterize the disease phenotype. Affected individuals displayed classic ocular signs of PA such as bilateral corneal opacities (Supplementary Fig. 1b), developmental glaucoma, iris–retina coloboma (except in individual 18), anterior segment dysgenesis and iridolenticular adhesions. Nystagmus was seen in all the affected individuals except individual 18. These syndromic features are present with variable degrees of penetrance in all affected individuals. No signs of any skeletal abnormalities, physical disabilities, cardiovascular diseases or mental retardation were seen in members of PKCC139.

### Genome-wide linkage analysis localized PA to chromosome 1p

To localize the disease phenotype, we performed a genome-wide linkage analysis with the ABI MD-10 genotyping panel. During the genome-wide scan, significant two-point logarithm of odds (LOD) scores (LOD>3) were obtained only with chromosome 1p makers (LOD score of 3.42 with marker D1S197 at *θ*=0). A recombination event in individual 11 at marker D1S2713 defined the proximal boundary, while the distal recombination event was marked by affected individual 9 at D1S2652 (Supplementary Fig. 1a). This defined the critical interval and placed it on chromosome 1p34.1–p32.3 in the 6.96 cM (10.95 Mb) interval flanked by markers D1S2713, proximally, and D1S2652, distally (Supplementary Fig. 1a).

### Sanger and next-generation whole-exome sequencing

The linked interval harbours an interesting candidate gene, *FOXE3*, a lens-specific transcription factor responsible for dominant forms of anterior segment dystrophy including PA and inherited primary aphakia^16^,^17^. Sanger sequencing of *FOXE3* in two affected individuals of family PKCC139 revealed a homozygous substitution (c.720C>A) leading to a premature stop codon at cysteine 240 (p.C240*). Sequencing of *FOXE3* in all available family members confirmed co-segregation of the mutation with PA (Supplementary Fig. 1a,c). The mutation was not found in 384 ethnically matched control chromosomes, in the 1000 genome data or the NHLBI exome variant server database. To exclude the possibility of an additional variant present within the critical interval contributing to the disease phenotype, we sequenced two affected individuals of PKCC139 through whole-exome sequencing. The exome analysis did not reveal any non-synonymous variants within the critical interval on chromosome 1p in either affected individual.

We had previously localized autosomal recessive isolated congenital cataracts in two families (PKCC009 and PKCC039) to chromosome 1p34 (ref. 18). Sequencing of *FOXE3* identified two novel homozygous mutations c.351C>G (p.N117K) and c.307G>A (p.E103K) in PKCC009 and PKCC039, respectively (Supplementary Fig. 1d–i). Both mutations segregated with the disease phenotype in their respective families and were absent in 144 control chromosomes of Pakistani decent and 24 control chromosomes of Saudi Arabian decent. Moreover, these variations were not present in the 1000 Genomes, NHLBI Exome Sequencing Project and the dbSNP databases, while evolutionary conservation analysis suggested that both amino acid residues (E103 and N117) are fully conserved in other FOXE3 orthologues. To exclude the possibility of an additional variant present within the critical interval responsible for the disease phenotype, we captured the exomes of two affected individuals from each of the two families and analysed them through next-generation sequencing. We systematically examined all the variants present within the critical interval of each family; however, we did not identify any variant(s) that would explain the causal phenotype.

### The C240* mutant FOXE3 localizes to the nucleus

To understand the physiological processes aberrantly regulated by the premature truncation of *FOXE3*, we first investigated the effect of C240* on the localization of FOXE3. *FOXE3* encodes a 319 amino acid transcription factor that localizes to the nucleus. Nuclear localization is guided by the nuclear localization signal (NLS), an amino acid sequence that marks a protein for import into the nucleus through nuclear transport machinery^19^. As the NLS of FOXE3 resides in the N terminus of the protein, we reasoned that the mutant FOXE3 would still successfully localize to the nucleus. To investigate the localization pattern of the mutant FOXE3, we transfected HeLa and HLE cells with plasmid constructs of the wild-type or mutant FOXE3–green fluorescent protein (FOXE3–GFP) fusion and tracked the localization of the fusion protein with either GFP fluorescence or anti-GFP antibody. The immunofluorescence tracking illustrated an indistinguishable pattern for the wild-type and mutant FOXE3 protein, both localizing to the nucleus in both cell lines (Supplementary Fig. 2a–l). These results indicate that the p.C240* mutation does not affect transportation and nuclear localization of the mutant FOXE3.

Immunofluorescence tracking illustrated an indistinguishable pattern for the wild-type and truncated FOXE3, confirming the nuclear localization of the mutant FOXE3 (Supplementary Fig. 2). We therefore reasoned that ocular phenotypes observed in PA patients may be due to the impairment or partial impairment of the transcriptional activities of FOXE3 resulting from diminished DNA-binding activity, especially in affected individuals of PKCC139, due to the absence of the 80-carboxy-terminal amino acids. Previously, Medina-Martinez *et al*.^13^ reported severe defects in proliferation and differentiation of lens epithelial cells in *Foxe3*^*−/−*^ knock-out (KO) mice. The *Foxe3*^*−/−*^ lenses could be an excellent resource to determine the downstream targets of FOXE3. However, the anterior lens epithelium cells prematurely cease to proliferate in *Foxe3*^*−/−*^ mice, leading to dramatically underdeveloped lenses resulting in complete aphakia^13^. Furthermore, the anterior lens epithelium fails to separate from the cornea while the retina shrinks and displays abnormal folding^13^. Therefore, we decided to express the mutant FOXE3 in a physiologically relevant human cell line and investigate the differentially expressed genes and proteins through next-generation sequencing-based transcriptome and MS-based proteome profiling, respectively.

### Transcriptome analysis of cells expressing C240* FOXE3

To identify the differentially expressed genes in physiologically relevant cells, we reasoned that an optimal approach would be to use cultured HLE cells. To eliminate any background resulting from endogenous *FOXE3*, it would be necessary to silence the endogenous expression. However, Wang *et al*.^20^ previously reported that short-hairpin RNA (shRNA)-mediated silencing of *FOXE3* significantly inhibits cell growth and induces G1-phase arrest in cultured HLE cells. Therefore, to preclude the possibility of confounding experimental interpretation, we opted to use cell lines negative for endogenous FOXE3 expression. We examined human embryonic kidney (HEK293FT) cells and HeLa cells for endogenous *FOXE3* expression using TaqMan probes and found no *FOXE3* detected in either cell line (*C*_T_>40 cycles).

**Figure 1:**
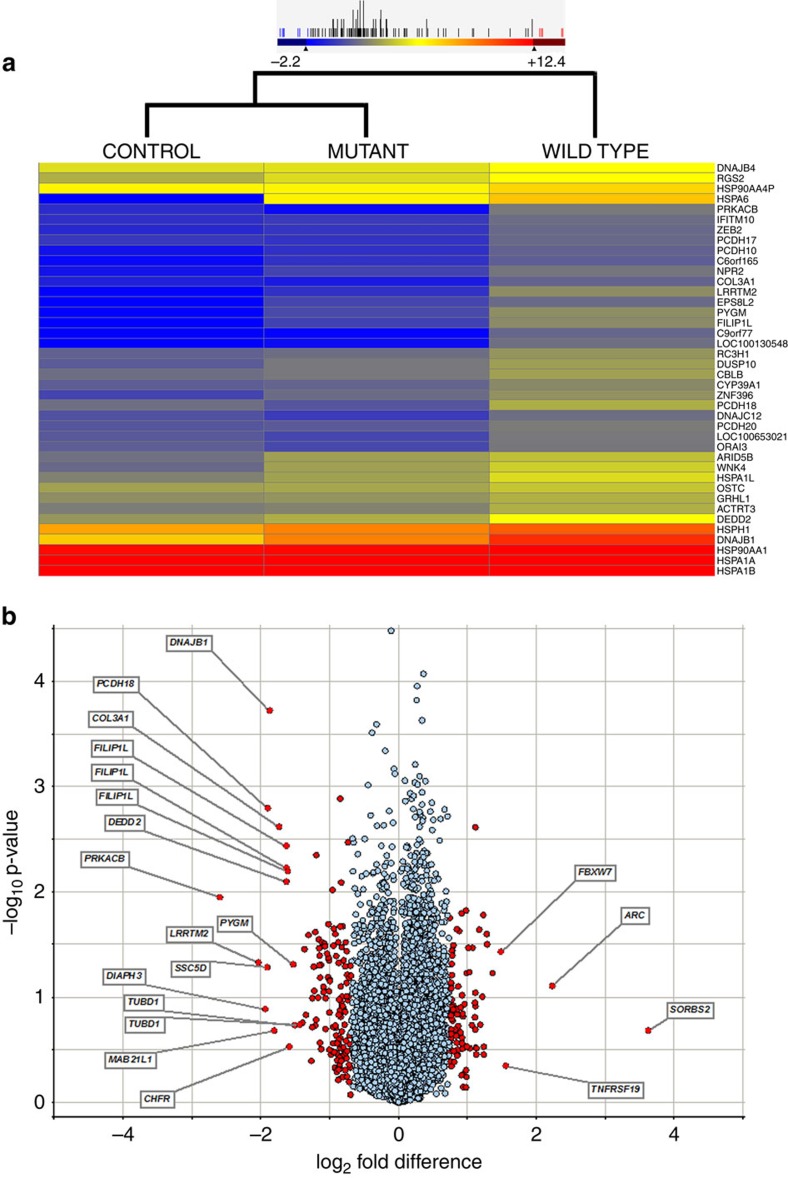
Illustration of the differentially regulated transcripts in HEK293 cells expressing mutant (p.C240*) FOXE3. (**a**) Clustering of transcripts that are downregulated in cells expressing the mutant *FOXE3*. The differentially expressed genes were selected on the basis of stringent criterion (*p*-value ≤0.05 (Student's *t*-test) and ≥1.5-fold change). The colour of each horizontal line represents the expression of mRNA in a given sample, whereas colour scale from blue to red indicates low to high gene expression. (**b**) Volcano plot representation of differentially expressed transcripts and their statistical significance. The fold changes are represented in log_2_ scale depicted on the *x*-axis, whereas the −log_10_ *p*-value is depicted on the *y*-axis (the use of -log values mean that transcripts with greater statistical significance are higher in the plot). The red circles represent genes that show 3 s.d. differential expression, while red circles with gene names represent differentially expressed transcripts at 6 s.d.

Next-generation sequencing of transcriptome libraries prepared from cell lines was performed on a HiSeq 2000 genome analyser. We used two lanes of the genome analyser, which generated 7.12 × 10^8^ reads for all samples, that is, eight biological replicates for the wild-type *FOXE3* expression, eight for the truncated *FOXE3* and eight for the negative control (Table 1). Mapping to the UCSC hg19 human genome reference yielded 4.64 × 10^8^ unique reads that were examined for differential expression (Table 1). A Pearson coefficient value (*R*^2^) of >0.98 was obtained for the respective replicates, suggesting a good correlation among biological replicates (Supplementary Fig. 3a). The gene expression comparison of the wild-type and mutant samples showed a higher Pearson coefficient value (*R*^2^) of 0.986, while *R*^2^ values of 0.978 and 0.975 were obtained by comparing gene expression in the control sample with mutant and wild-type samples, respectively (Supplementary Fig. 3b). To investigate the differential expression, we first subtracted the total reads of the normalized control from the reads of the normalized wild-type FOXE3 and that of the reads from the truncated FOXE3 samples to reduce the background. Subsequently, we compared the transcript levels (after background subtraction) of cells expressing the wild-type and truncated FOXE3. This analysis revealed 48 differentially expressed transcripts, which included 40 downregulated and 8 upregulated genes in cells expressing the truncated FOXE3 (Fig. 1a and Supplementary Table 1). Of these, we have validated expression of 37 genes in the mouse lens transcriptome (Supplementary Table 1). We identified nine HSP genes, *DNAJB1, DNAJB4, HSPA1A, HSPA1B, HSP90AA1, HSPA1L, DNAJC12, HSPH1* and *HSPA6*, which were all downregulated in cells expressing the truncated FOXE3 (Supplementary Table 1).

ArrayStar normalized linear RPKM values were used to determine differential gene expression between mutant and wild-type samples, and the results were exported to Spotfire DecisionSite for further evaluation (please see Methods). These analyses yielded 19,426 valid comparisons representing 13,510 unique genes. The s.d. was determined for each mutant to wild-type log2-fold change in transcript concentration as deviation from its mean of 0 or no change. A 3 s.d. cutoff representing 1.62-fold change revealed a subset of 225 unique genes (132 downregulated and 93 upregulated) that were differentially regulated in response to the expression of C240* FOXE3. We further increased the stringency by applying a 6 s.d. cutoff to avoid any false positive targets and re-analysed the transcriptome data. The increased stringency narrowed down the number of differentially expressed genes to 17, of which 13 were downregulated while 4 were upregulated (Fig. 1b). Among the 17 differentially expressed genes, only *Diaph3* was not present in the mouse lens transcriptome.

Most of the algorithms used for bioinformatics analysis have inherent limitations, and no single software package is perfect for all situations. To gain confidence in our analysis, we re-analysed our transcriptome data using the TopHat/DESeq. Differential expression analysis revealed a total of 41 differentially expressed transcripts including 31 transcripts that were downregulated and 10 transcripts that were upregulated in cells expressing the truncated FOXE3 (Supplementary Table 2). Of these, 25 genes are present in the mouse lens transcriptome (Supplementary Table 2). We identified five HSP genes (*DNAJB1*, *HSPA4L*, *HSPH1*, *HSPE1* and *HSPA6*) that were downregulated in cells expressing the truncated FOXE3 (Supplementary Table 2).

Taken together, a total of 106 genes showed a differential expression pattern that was identified using 3 different algorithms. Interestingly, only three genes were identified in all three analyses and more importantly, all three were downregulated in cells expressing the truncated FOXE3. These were *DNAJB1, PYGM* and *COL3A1*.

### Proteome profiling of cells expressing C240* FOXE3

Protein profiling through MS provides an excellent platform to investigate differentially expressed proteins with reproducible accuracy. Labelling with multiplexed isobaric reagents allowed us to simultaneously compare the effect of wild-type and mutant FOXE3 on the overall proteome in a high-throughput tandem MS (MS/MS) analysis with biological replicates. The 8PLEX iTRAQ labelling strategy yielded 3,781 unique proteins (comprising 17,022 unique peptides) from 26,130 peptide spectra matches (showing <30% interference) from a total of 28,684 peptide-spectrum matches when 1% false discovery rate (FDR) cutoff and top rank peptide for the spectrum were considered. On the other hand, 8PLEX tandem mass tag reagent (TMT) labelling experiment identified 3,315 unique proteins (comprising 12,021 unique peptides) from 17,435 peptide spectra matches (showing <30% interference) from a total of 17,959 peptide spectra matches. In iTRAQ, 7 and 8 unique peptides from 9 and 15 unique spectra were used for the quantification of DNAJB1 and FOXE3, respectively (Supplementary Data 1). For TMT proteome profiling, 7 and 8 unique peptides from 9 and 33 unique spectra were used for the quantification of DNAJB1 and FOXE3, respectively (Supplementary Data 2).

The proteome profiling revealed equal expression of both the wild-type and mutant FOXE3. The annotated MS/MS spectra of the peptide (GEAAPTPAPGPGR) from the N-terminal region showed similar expression in cells transfected with wild-type and mutant FOXE3 and was not detectable (dotted lines) in control cells (Supplementary Fig. 4a). Conversely, the quantification of the C terminus peptide (QPGFASGLER) showed complete lack (dotted lines) in cells transfected with mutant FOXE3 and control cells (Supplementary Fig. 4b).

**Figure 2:**
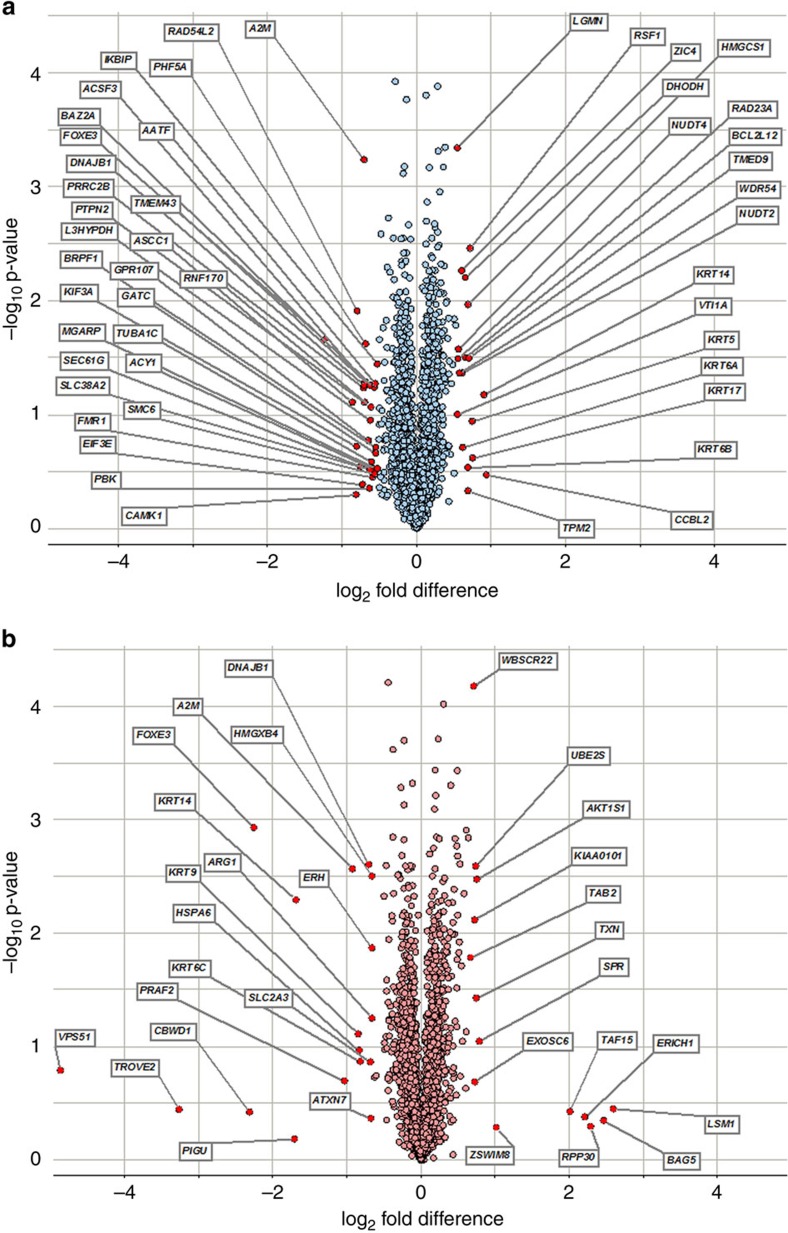
Volcano plot of differentially regulated proteins in HEK293 cells expressing mutant (p.C240*) FOXE3. (**a**) The iTRAQ protocol identified 48 differentially expressed proteins, of which 29 were downregulated and 19 upregulated in cells expressing mutant FOXE3. (**b**) The TMT protocol revealed a total of 31 proteins that were differentially expressed including 17 downregulated and 14 upregulated in cells expressing mutant FOXE3. Note: the fold changes are represented in log_2_ scale depicted on the *x*-axis and the −log_10_ *p*-value is depicted on the *y*-axis (the use of −log values means that proteins having greater statistical significance are higher in the plot). The red circles in both plots represent proteins that show 3 s.d. differential expression.

For both iTRAQ and TMT platforms, the s.d. was determined for each mutant to wild-type log2-fold change in protein concentration as a deviation from its mean of 0 or no change. A threshold of 3 s.d. was considered to establish differential protein expression between mutant and wild-type cells. The iTRAQ protocol identified 48 differentially expressed proteins, of which 29 were downregulated in cells expressing mutant FOXE3 and 19 upregulated with a 3 s.d. cutoff value, representing a 1.45-fold change (Fig. 2a). On the other hand, the TMT protocol revealed a total of 31 proteins that were differentially expressed, including 17 downregulated in cells expressing mutant FOXE3 and 14 upregulated with a 3 s.d. cutoff value, representing a 1.62-fold change (Fig. 2b). Of the 2,556 proteins that appear on both platforms, 3 proteins manifested a 3 s.d. differential regulation in both protocols: A2M and DNAJB1 were downregulated in both protocols while KRT14 was downregulated in TMT and upregulated in iTRAQ.

### *DNAJB1* is the sole candidate identified in both screens

Next-generation RNA sequencing (RNA-Seq) and MS-based protein profiling are complementary approaches, each of which has its inherent advantages, and, therefore, we adopted both techniques to identify transcripts and/or proteins that are differentially expressed in the cells expressing the WT and p.C240*-truncated FOXE3. The RNA-Seq analysis identified three genes, *DNAJB1, COL3A1* and *PYGM* that were downregulated −1.87, −1.74 and −1.53 log2-fold, respectively, in the cells expressing the WT versus the p.C240*-truncated FOXE3 (Fig. 1b). Likewise, protein sequencing (iTRAQ and TMT) identified three proteins showing consistent changes in expression: A2M was downregulated −0.71 (for iTRAQ) and −0.93 (for TMT) and DNAJB1 was downregulated −0.71 log2-fold (for both iTRAQ and TMT), while KRT14 showed 0.90 log2-fold upregulation (for iTRAQ) and −1.69 log2-fold downregulation (for TMT) (Fig. 2a,b). Notably, *DNAJB1* was the only differentially expressed candidate identified in both the transcriptome and proteome screens.

### *Dnajb1* is expressed in the developing mouse lens

Our findings suggest *DNAJB1*, a transcriptional target of FOXE3 that is differentially expressed in response to the non-sense mutation, to be responsible for PA. To understand the physiological relevance of our candidate in the pathogenesis of PA, we sought evidence of *FOXE3* and *DNAJB1* relative expression patterns in the anterior segment of the eye, especially at early developmental stages. We investigated the expression of *Foxe3* and *Dnajb1* in mouse lenses, and detected expression as early as embryonic day 12.5 (Supplementary Fig. 5). Furthermore, we observed a reciprocal expression pattern of *Foxe3* and *Dnajb1* in the developing mouse lens (Supplementary Fig. 5).

### FOXE3 binds to the *DNAJB1* promoter region

**Figure 3:**
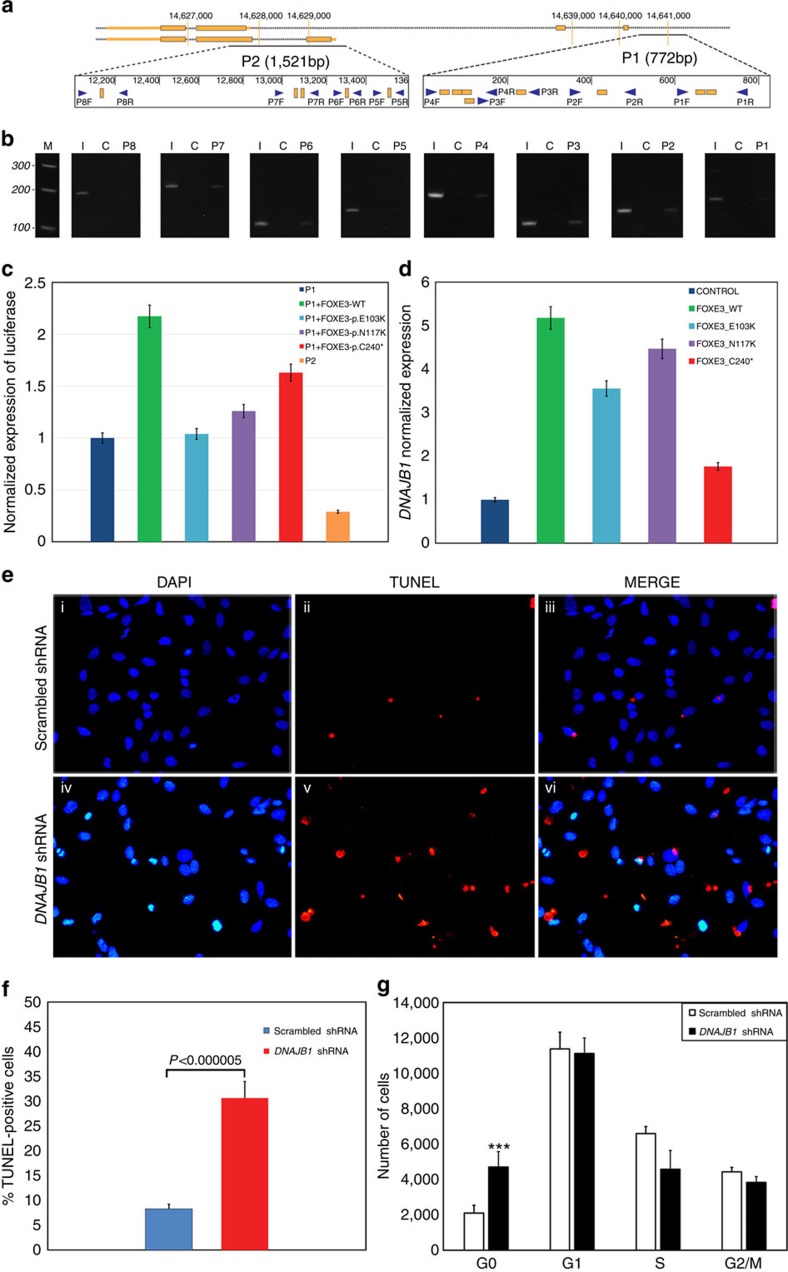
*DNAJB1* is a downstream transcriptional target of FOXE3 and is indispensable for the cell cycle. (**a**) Schematic illustration of the 5′ UTR of *DNAJB1* with golden boxes indicating potential FOXE3-binding sites predicted by ProSuite algorithms. Blue arrows, designated 1–8 F and 1–8 R, represent eight primer pairs used for amplifying regions of interest. (**b**) Amplification of specific segments in the 5′ UTR of *DNAJB1* using pull-down DNA as the template in the amplification reaction. (**c**) HEK293FT cells were transiently transfected with the *DNAJB1*-GLuc-SEAP vector and an expression vector harbouring wild-type or mutant (p.E103K, p.N117K and p.C240*) FOXE3 alleles. Error bars represent percent deviation in each data set of luciferase activity normalized against SEAP performed in seven independent replicates for each condition. (**d**) HEK293FT cells were transfected with the wild-type or mutant FOXE3-expressing plasmids in a pT-Rex-DEST30 vector and expression levels of *DNAJB1* were quantitated with TaqMan assays. Error bars represent s.d. in each data set of the *DNAJB1* expression normalized against *GAPDH*, analysed in six independent replicates. (**e**) HLE cells were transfected with either scrambled (i–iii) or *DNAJB1* shRNA (iv–vi), and the increase in apoptotic cell population in response to shRNA-targeting *DNAJB1* was assessed 48 h post transfection. (**f**) Increased apoptosis resulting from downregulation of *DNAJB1* was estimated through flow cytometry of TUNEL stained cells 48 h post transfection of *DNAJB1* shRNA. The loss of *DNAJB1* results in increased apoptosis in HLE cells resulting in a threefold increase in TUNEL-positive cells compared with the control cells transfected with scrambled shRNA (*P*<0.000005; 2-tailed student's *t*-test). Error bars represent s.d. in each data set analysed in four independent replicates. (**g**) The effect of the downregulation of *DNAJB1* on the G0 population was examined through flow cytometry of propidium iodide-stained cells 48 h post transfection of *DNAJB1* shRNA. The *DNAJB1* shRNA-transfected cells exhibited a significant increase in the G0 population as compared with the control cells transfected with scrambled shRNA. Error bars represent s.d. in each data set analysed in four independent replicates. ‘***' indicates *P*<0.00001 (two-tailed student's *t*-test).

Taken together, our RNA-Seq and proteome analysis suggest a regulatory mechanism of *DNAJB1* through FOXE3 transcriptional activity. One possibility is that FOXE3 directly regulates *DNAJB1* by binding to its promoter region while a second is that FOXE3 might mediate control by acting through other transcription factors and/or enhancers of transcriptional activity. We sought additional evidence to differentiate between the above-mentioned possibilities. *In silico* analysis identified multiple FOXE3-binding sites in the 5′ untranslated region (UTR) of *DNAJB1*. We further investigated these binding sites, employing chromatin immunoprecipitation (ChIP), to confirm the results of *in silico* analysis. Specific-sized PCR products were identified in amplification reactions that included FOXE3 antibody pull-down as the DNA template. These findings confirm that FOXE3 binds to the 5′ UTR of *DNAJB1* and modulates the transcriptional activity of *DNAJB1* (Fig. 3a,b). To confirm the results of ChIP analysis, we designed luciferase reporter constructs harbouring two DNA fragments from the 5′ UTR sequence of *DNAJB1*, a 772-bp fragment (designated as P1) and a 1,521-bp fragment (designated as P2). Both of these fragments were examined for FOXE3 binding in the ChIP analysis. The expression of wild-type FOXE3 showed a twofold higher luciferase signal in the presence of P1 DNAJB1-GLuc-SEAP vector (Fig. 3c). Subsequently, we evaluated the deficiency of FOXE3 mutant alleles (E103K, N117K and C240*). Our results suggest that all three mutant FOXE3 proteins exhibit lower transcriptional activity for the putative P1 FOXE3-binding sites (Fig. 3c). Also, we used a promoter-less or negative control GLuc-SEAP vector alone and in combination with wild-type FOXE3 to measure the luciferase activity in HEK293 cells. Consistent with previous results, expression of wild-type FOXE3 along with P1 DNAJB1-GLuc-SEAP vector showed greater than threefold higher luciferase activity compared with the negative control vector (Supplementary Fig. 6). We also observed approximately twofold higher luciferase signal when expressing the P1 DNAJB1-GLuc-SEAP vector compared with the negative control vector (Supplementary Fig. 6). Concordantly, lower luciferase signals were observed for the P1 DNAJB1-GLuc-SEAP vector in the presence of FOXE3 mutants (Supplementary Fig. 6).

### Mutant *FOXE3* alleles compromise *DNAJB1* expression

Our observations strongly indicate that we have identified *DNAJB1* as a downstream target of FOXE3. Subsequently, we performed a reciprocal experiment by examining the relative expression levels of *DNAJB1* in HEK293FT cells expressing either the wild-type, truncation mutant (p.C240*) or two missense variants of FOXE3 (p.E103K and p.N117K). Overexpression of FOXE3 identified a threefold higher expression of *DNAJB1* in cells transfected with wild-type FOXE3 when compared with cells expressing the mutant truncated *FOXE3*, which was consistent with the RNA-Seq and MS analysis (Fig. 3d). The quantitative real-time PCR (qRT-PCR) confirmed the differential expression of *DNAJB1* in response to all three mutant *FOXE3* alleles; however, the decrease in DNAJB1 expression seen with transfection of the E103K and N117K alleles showed different levels for the loss of activity in the two experiments (Fig. 3d). The point mutations p.E103K and p.N117K are located within the putative DNA-binding domain of FOXE3 and, therefore, most likely alter the affinity of this transcription factor for DNA. The deficiency in the DNA-binding activity of the mutant protein theoretically could be compensated by overexpression of the mutant protein. Although the mutant proteins are overexpressed in both experiments, the promoter sequence (772 bp) utilized in the luciferase assay is smaller than the endogenous *DNAJB1* promoter and lacks any regulatory sequences present in the 5′ UTR of *DNAJB1*. Collectively, the lack of an optimal promoter and the DNA-binding deficiency are the most plausible explanations of the distinctive functional activities of the missense mutant proteins in the two *in vivo* experiments.

We repeated the luciferase reporter assay in HLE cells to reproduce the results in a physiologically relevant system. The expression of wild-type *FOXE3* caused significant alteration in shape and eventually death in HLE cells, consistent with previously published reports of ectopic expression of *Foxe3* in the mouse lens^20^,^21^.

### Knockdown of *DNAJB1* in HLE cells results in G0/G1 arrest

Next we examined a more physiologically relevant model that mimicked haploinsufficiency by knocking down *DNAJB1* in HLE cells. We achieved a 50% reduction in the relative expression of *DNAJB1* along with a significant loss of confluence 48 h post transfection compared with the scrambled shRNA-transfected cells (Supplementary Fig. 7). *DNAJB1* shRNA induced a 3.5-fold reduction in HLE cell number 48 h post transfection (*P*<0.005) compared with scrambled shRNA-transfected cells. We examined these cells with the Terminal deoxynucleotidyl transferase dUTP Nick End Labelling (TUNEL) assay, which illustrated more apoptotic nuclei in cells transfected with shRNA targeting *DNAJB1* (Fig. 3e). In parallel, we detected increased apoptosis (threefold; *P*<0.000005) in HLE cells by knocking down *DNAJB1* through shRNA using flow cytometry (Fig. 3f). Flow cytometry analysis of propidium iodide (PI)-stained HLE cells revealed a twofold higher (*P*<0.000001) increase in the G0 population of *DNAJB1* downregulated cells compared with HLE cells transfected with scrambled shRNA (Fig. 3g). Previously, Wang *et al*.^20^ reported that shRNA-mediated knockdown of *FOXE3* in HLE cells resulted in an increased G0 population. Taken together, these results confirm that *DNAJB1* is a downstream target of FOXE3.

### *In vivo* modelling of *DNAJB1* recapitulates signs of PA

To examine the *in vivo* functional relevance of DNAJB1 to PA, we targeted the endogenous expression of the zebrafish orthologue of *DNAJB1*. We reasoned that if *DNAJB1* is one of the downstream targets of FOXE3, then suppression of *dnajb1* in zebrafish embryos should recapitulate some, if not all, of the cardinal signs of PA. To test our hypothesis, we decided to knock down *dnajb1* in zebrafish and examined both ocular and extra-ocular phenotypes. Zebrafish have two orthologues of *DNAJB1*, *dnajb1a* and *dnajb1b*. Interestingly, *dnajb1a* is expressed ubiquitously while expression of *dnajb1b* appears to be predominantly localized to the lens (http://zfin.org). It is important to note that human *DNAJB1* is expressed ubiquitously (http://research-public.gene.com/Research/genentech/genehub-gepis/genehub-gepis-search.html), which is similar to *dnajb1a* in zebrafish. Therefore, to more accurately recapitulate human phenotype mimicking symptoms of PA, we decided to target *dnajb1a* because its expression profile is most similar to *DNAJB1*. Moreover, knockdown of the lens-specific *dnajb1b* would preclude the possibility of identifying extra-ocular phenotypes.

First, we injected 100 embryos with a splice-blocking morpholino (sbMO) targeted against *dnajb1a*. We used a MO designed to bind to the splice acceptor site, (Supplementary Fig. 8a), which would disrupt wild-type *dnajb1a* mRNA splicing (Supplementary Fig. 8b) by either retaining intron 1 (Supplementary Fig. 8c) or excising exon 2 along with introns 1 and 2 (Supplementary Fig. 8d). Targeting of *dnajb1a* expression resulted in a significant reduction of the wild-type spliced mRNA in embryos at 12 and 24 h post fertilization compared with the controls (*P*<0.0001), assessed by qRT-PCR (Supplementary Fig. 8e). To differentiate between the two splicing scenarios, we tested the *dnajb1a* morphant mRNA with primers on exons 1 and 2, which did not yield any specific PCR product, thereby confirming the effect of the *dnajb1a* morpholino. In contrast, the control complementary DNA (cDNA) generated a mean *C*_T_ of 25 cycles (*C*_T_ for β-actin was 22 cycles). The unaltered expression of *dnajb1b* in *dnajb1a* morphants (Supplementary Fig. 8e) further confirmed the specificity of the *dnajb1a* sbMO.

**Figure 4:**
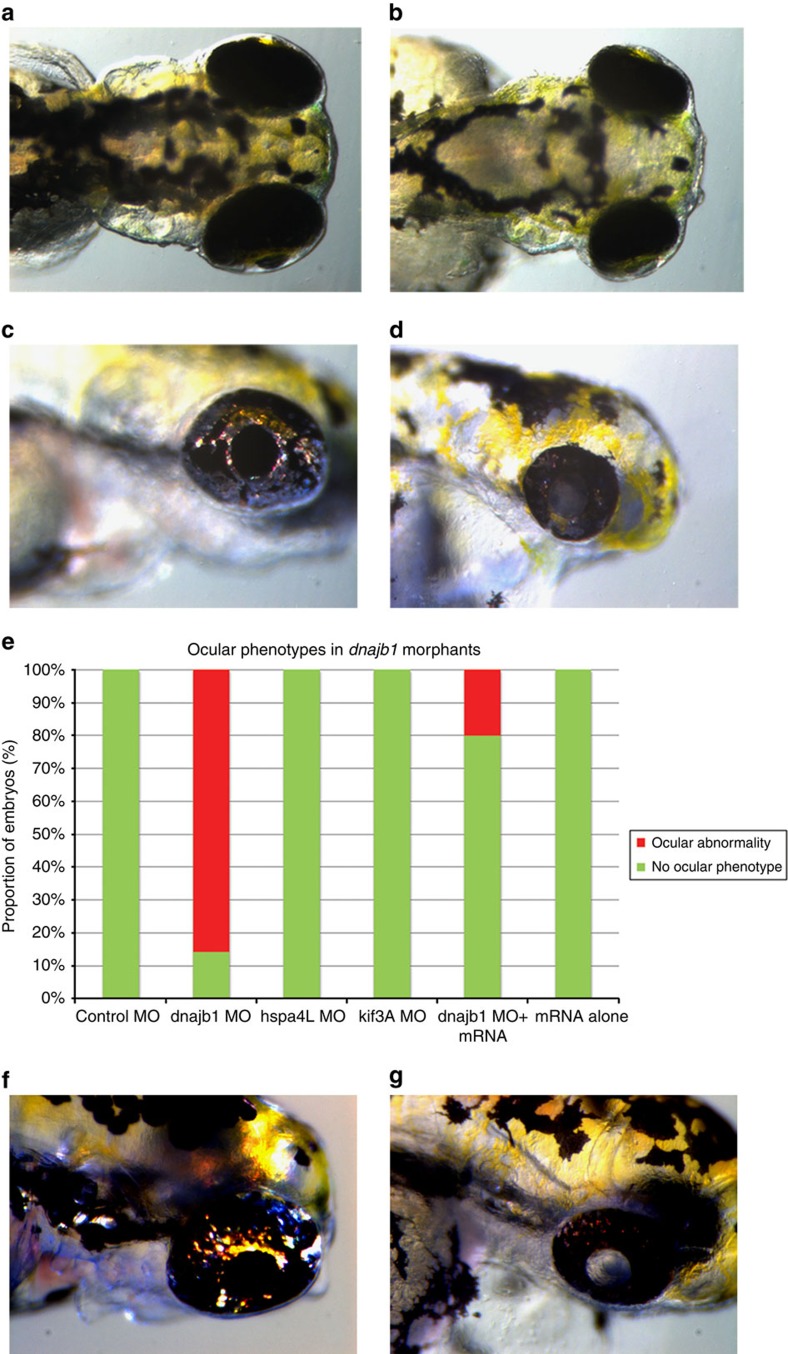
*In vivo* modelling reveals broader developmental defects and underdeveloped cataractous lenses in *dnajb1*a morphants. *dnajb1a* zebrafish morphants recapitulate PA symptoms. Dorsal view of (**a**) control morphant and (**b**) *dnajb1a* morphant embryos suggest a relatively compact lens observed at 4 days post fertilization as a result of *dnajb1a* MO. Lateral view of (**c**) control morphant and (**d**) *dnajb1a* morphant embryos showing broader eye development defects including underdeveloped cataractous lenses in *dnajb1a* MO-injected embryos. (**e**) Quantification of the compact lens phenotype in the *dnajb1a*, *hspa4l* and *kif3a* morphants and rescue of morphant phenotype with zebrafish orthologue of *dnajb1a* mRNA. Note: *kif3a* MO did not show any ocular phenotype; however, the morphants exhibited morphological and developmental defects, such as body curvature, consistent with the ciliary phenotypes. Lateral view of (**f**) control morphant and (**g**) *dnajb1b* morphant embryos illustrate a relatively compact cataractous lenses observed at 4 days post fertilization as a result of *dnajb1b* translation-blocking MO.

After confirming the MO-dependent splicing defect, we re-injected embryos with the sbMO and examined for ocular, as well as non-ocular phenotypes. The dorsal view of the embryos observed at 4 days post fertilization (dpf) suggested the morphants had relatively smaller eyes when compared with controls (Fig. 4a,b), whereas the lateral view suggested broader eye developmental defects, including an underdeveloped lens with total cataracts in the morphants lenses, recapitulating the symptoms of PA (Fig. 4c,d).

**Figure 5:**
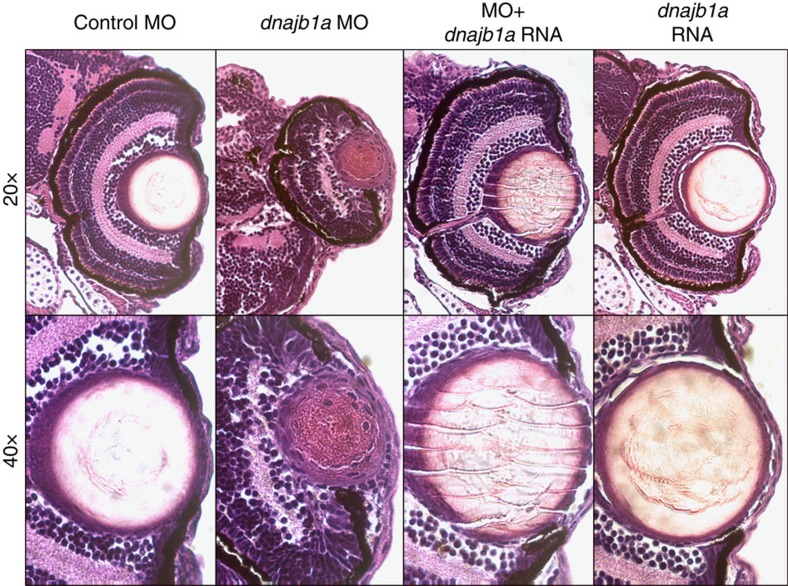
Histological evaluation of the developmental defects in the anterior segment of *dnajb1a* morphant eyes. Zebrafish embryos injected with the dnajb1a sbMO morpholino (5 ng), or the morpholino plus *dnajb1a* mRNA (150 pg) or *dnajb1a* mRNA. Embryos were fixed 4 days post fertilization in 4% paraformaldehyde and embedded in paraffin. Sections (5 μm) were stained with haematoxylin and eosin and visualized under a microscope for histological evaluation.

To further confirm that the ocular phenotype is a direct result of the *dnajb1a* knockdown, we rescued the morphant phenotype by co-injecting *in vitro*-transcribed *dnajb1a* mRNA with the sbMO. Co-injection of 150 pg of *dnajb1a* mRNA resulted in a complete rescue of the morphants ophthalmic phenotypes (80% of embryos, *n*=100–150: Fig. 4e). The morpholinos against targets (*hspa4l* and *kif3a*) identified either in the transcriptome or the proteome screen, but not both, resulted in an unidentifiable ocular phenotype (Fig. 4e). The histological examination of *dnajb1a* morphants at 4 dpf showed smaller eyes with a somewhat elongated lens, deteriorated cornea and distorted angle mesenchyme compared with the controls (Fig. 5). Co-injecting *in vitro*-transcribed *dnajb1a* mRNA rescued the morphant phenotype (Fig. 5), while *dnajb1a* mRNA alone did not result in any ocular phenotype (Fig. 5).

The above *in vivo* data confirm both the efficacy and specificity of the *dnajb1a* MO. Nevertheless, we do recognize that the use of a single morpholino may not be sufficient to adequately demonstrate the particular nature of the MO-induced ocular defects. Therefore, we designed two additional MOs, a sbMO and a translation-blocking MO (tbMO) targeting *dnajb1b*. First, we injected 100 embryos with a sbMO targeted against *dnajb1b*. The sbMO binds to the splice donor site (Supplementary Fig. 9a) and should disrupt wild-type *dnajb1b* mRNA splicing (Supplementary Fig. 9b) by retaining intron 1 (Supplementary Fig. 9c). Targeting *dnajb1b* expression resulted in a significant reduction of the wild-type spliced mRNA in embryos at 12  and 24 h post fertilization compared with the controls (*P*<0.0003), assessed by RT-PCR (Supplementary Fig. 9d). As observed in *dnajb1a* morphants, the expression of *dnajb1a* was not significantly different in *dnajb1b* morphants (Supplementary Fig. 9d), which once again confirmed the specificity of the *dnajb1b* sbMO.

The *dnajb1b* morphants displayed an equal proportion of mild, and severely affected morphology. Histological analysis of the mild phenotype embryos showed protruding lenses in the morphants that were relatively small in size compared with the control embryos (Supplementary Fig. 10). In contrast, the severely affected embryos had smaller eyes with a somewhat elongated/bulged lens compared with the controls (Supplementary Fig. 10). In parallel, we injected 10 ng of tbMO that resulted in total cataracts in the morphants lenses (Fig. 4f,g), recapitulating the symptoms of PA.

**Figure 6:**
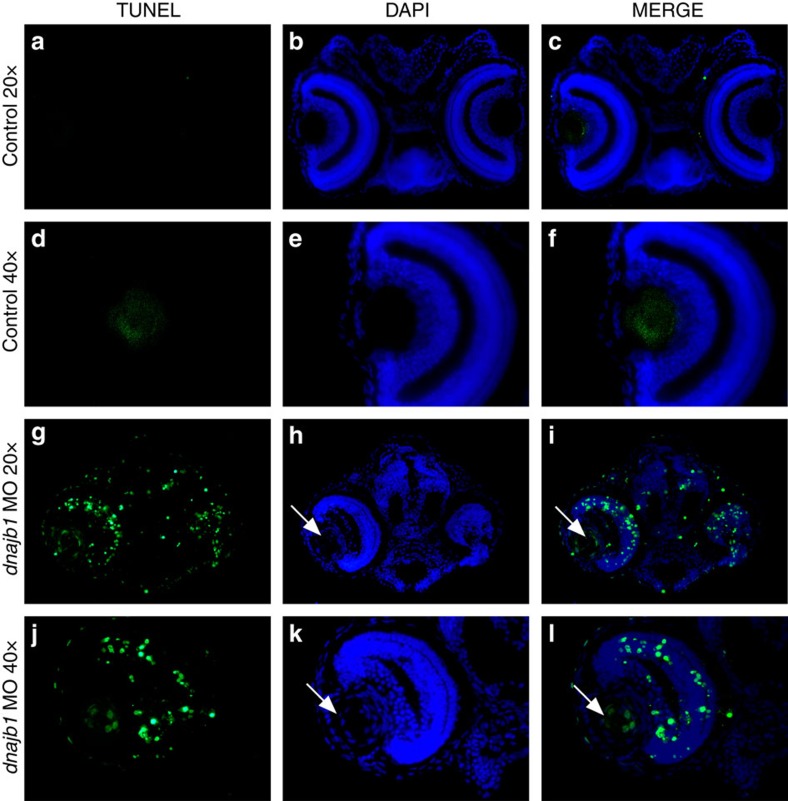
*dnajb1* morphants display significant apoptosis in the eye. TUNEL staining of control embryo sections (**a**–**f**) and *dnajb1* morphants (**g**–**l**) injected with the dnajb1a sbMO morpholino (5 ng). The *dnajb1a* morphants show significantly increased apoptotic nuclei throughout the eye compared with the control embryo, which was completely devoid of TUNEL-positive nuclei. The morphant lens extends out of the retina and is fused to the cornea with an increased number of nuclei as compared with the control (white arrows, **h**,**k**) and is characterized by the presence of apoptotic nuclei in the centre of the lens (white arrows, **i**,**l**), suggesting an abnormal lens development. Note: the control embryo section image was acquired at a higher exposure time compared with the morphant to produce a background.

The persistence of nuclei in the morphant lenses suggests an incomplete differentiation of lens fibre cells, a possible defect in the formation of an organelle-free zone. Since the knockdown of *DNAJB1* in HLE cells resulted in increased apoptosis, we tested if a similar defect could be observed *in vivo*. TUNEL staining of 4 dpf zebrafish embryo sections further revealed specific localization of the TUNEL-positive nuclei. As shown in Fig. 6, cells in the centre of the morphant lens and the retinal cells showed significantly increased apoptosis when compared with normal embryos, where only one to three TUNEL-positive cells were found in the eye.

## Discussion

Here we demonstrate that *DNAJB1* is a downstream transcriptional target of *FOXE3* through a combination of transcriptome and proteome analysis that identified a novel pathway, indispensable for the development of the anterior segment of the eye and especially for the ocular lens. DNAJB1, a member of the heat shock 40 kDa protein family has been associated with several cellular processes including the proteasome pathway, endoplasmic reticulum (ER) stress response, and more recently, cancer progression^22^,^23^,^24^. However, to the best of our knowledge, this is the first report establishing the crucial role of DNAJB1 in the development and maintenance of lens transparency.

The DnaJ/Hsp40 proteins are known to interact with chaperone proteins, Hsp70s, to stimulate their ATPase activity, which is essential for assisting unfolded proteins to reacquire their native conformation. In lens fibres, which are non-dividing cells with little protein turnover, the chaperone activity of HSPs would be indispensable to maintain the properly-folded confirmation of cellular proteins. Nevertheless, it is unlikely that the chaperone activity of DNAJB1 is the cause of these cataracts, as CRYAA, CRYAB and HSP90 are expressed at high levels in the lens and should be able to compensate for the loss of chaperone action.

We show that FOXE3 regulates *DNAJB1* expression while a previously published promoter analysis of Hsp40, a human homologue of bacterial DnaJ, revealed that its promoter contains a consensus heat shock element DNA-binding sequence for heat shock factor 1 (HSF1)^25^. HSF1 is expressed in developing ocular lenses^26^, and we, therefore, cannot rule out the possibility that HSF1 and/or other yet unidentified transcription factors also regulate expression of DNAJB1 in the ocular lens.

Interestingly, DNAJB1 has been shown to destabilize PDCD5 and suppress p53-mediated apoptosis^27^. Moreover, Qi *et al*.^28^ showed that DNAJB1 stabilizes MDM2, resulting in increased MDM2-mediated ubiquitination and degradation of p53. In parallel, the first evidence of the possible role for DNAJB1 in autophagy was presented by Behrends *et al*.^29^, who showed that WIPI2 associates with ATG2A indirectly through DNAJB1 and that depletion of WIPI2 led to reduced numbers of autophagosomes. Subsequently, it was shown that WIPI2, an Atg18 homologue, is required for LC3 lipidation and that WIPI2 forms punctate structures that co-localize with LC3 on early autophagosomal structures but not on mature autophagosomes^30^,^31^. Taken together, these data suggest that DNAJB1 is associated both with apoptosis and autophagy.

Our data show that downregulation of *DNAJB1* results in underdeveloped cataractous lenses, suggesting a crucial role of DNAJB1 in the development of the ocular lens that is not compensated by the extremely high levels of chaperones αA- and αB-Crystallin proteins in the lens. This suggests that loss or inhibition of the interaction of DNAJB1 with HSP70 chaperone proteins to stimulate their ATPase is not the cause of developmental abnormalities in the zebrafish lens. We propose that DNAJB1 has an essential role in the development of the ocular lens, especially the differentiation of fibre cells in which the cellular organelles are removed through autophagy, resulting in enucleated fibre cells and the transparent crystalline lens essential for focusing light on the retina. Consistent with this hypothesis, loss of autophagy has previously been implicated in congenital cataracts^32^. On the other hand, the absence or decrease of DNAJB1 leads to a cataractous lens with persistent nuclei that eventually are lost through apoptosis.

In conclusion, we have identified *DNAJB1* as a downstream target of FOXE3 through combined transcriptional and proteomic studies that were subsequently validated through both *in vitro* and *in vivo* approaches. To the best of our knowledge, this is the first report establishing the crucial role of *DNAJB1* in the development and maintenance of lens transparency.

## Methods

### Subjects and clinical ascertainment

A large familial case, PKCC139, with multiple members presenting symptoms consistent with Peters anomaly was recruited to participate in a collaborative study to investigate the genetic basis of anterior segment anomalies. Two additional familial cases (PKCC009, and PKCC039) reported previously^18^, were also included in the current study. Institutional Review Board (IRB) approval was obtained from the Johns Hopkins University School of Medicine, Baltimore MD, the National Institutes of Health, Bethesda MD, the King Khaled Eye Specialist Hospital, Riyadh Saudi Arabia, and the National Centre of Excellence in Molecular Biology, Lahore Pakistan. The participating subjects gave informed consent consistent with the tenets of the Declaration of Helsinki.

A detailed medical history was obtained by interviewing family members, and all participating individuals underwent a thorough ophthalmic examination including slit-lamp microscopy. A small aliquot of blood sample was collected from all participating members of the family and stored in 50 ml Sterilin Falcon tubes (BD Biosciences, San Jose, CA) containing 400 μl of 0.5 M EDTA. Blood samples were placed at −20 °C for long-term storage.

Genomic DNA was extracted from white blood cells. Briefly, 10 ml of the blood sample was mixed with 35 ml of TE buffer (10 mM Tris-HCl, 2 mM EDTA, pH 8.0), and the TE-blood mixture was centrifuged at 2,000*g* for 20 min. The red blood cells were discarded, and the pellet was re-suspended in 35 ml of TE buffer. The TE washing was repeated two to three times and the washed pellet was re-suspended in 2 ml of TE buffer. Next, 6.25 ml of protein digestion cocktail (50 μl (10 mg ml^−1^) of proteinase K, 6 ml TNE buffer (10 mM Tris-HCl, 2 mM EDTA, 400 mM NaCl) and 200 μl of 10% sodium dodecyl sulfate) was added to the re-suspended pellets and incubated overnight in a shaker (250 r.p.m.) at 37 °C. The digested proteins were precipitated by adding 1 ml of 5 M NaCl, followed by vigorous shaking and chilling on ice for 15 min. The precipitated proteins were pelleted by centrifugation at 2,000*g* for 20 min and removed. The supernatant was mixed with equal volumes of phenol/chloroform/isoamyl alcohol (25:24:1), and the aqueous layer containing the genomic DNA was carefully collected. The DNA was precipitated with isopropanol and pelleted by centrifugation at 3,500*g* for 15 min. The DNA pellets were washed with 70% ethanol and dissolved in TE buffer.

### Genome-wide linkage analysis and Sanger sequencing

The Applied Biosystems MD-10 linkage mapping panels (Applied Biosystems, Foster City, CA) were used to complete a genome-wide scan. Multiplex PCR was performed in a 5 μl mixture containing 40 ng of genomic DNA, 0.5 μl of 10 μM fluorescent-labelled primer pairs, 0.5 μl of 10 × PCR Buffer (100 mM Tris HCl (pH 8.4), 400 mM NaCl, 15 mM MgCl_2_, 2.5 mM Spermidine), 250 μM dNTP mix, and 0.2 U Taq DNA Polymerase (New England Biolabs, Cat # M0320S). Initial denaturation was performed for 5 min at 95 °C, followed by 10 cycles of 15 s at 94 °C, 15 s at 55 °C and 30 s at 72 °C and then 20 cycles of 15 s at 89 °C, 15 s at 55 °C and 30 s at 72 °C. The final extension was performed for 10 min at 72 °C. PCR products were mixed with a loading cocktail containing HD-400 size standards (Applied Biosystems) and resolved in an Applied Biosystems 3100 DNA Analyser. Genotypes were assigned using the Gene Mapper software from the Applied Biosystems.

Linkage analysis was performed with alleles of PKCC139 obtained through the genome-wide scan using the FASTLINK version of MLINK from the LINKAGE Program Package (provided in the public domain by the Human Genome Mapping Project Resources Centre, Cambridge, UK)^33^,^34^. PA was analysed as a fully penetrant autosomal recessive trait with an affected allele frequency of 0.001.

Individual amplicons of *FOXE3* were amplified by PCR using primer pairs designed by the primer3 program (Supplementary Table 3). PCR reactions were completed in 10 μl volumes containing 20 ng of genomic DNA, 1 μl of 10 μM of the forward and reverse primers, 1 μl of 10 × PCR buffer (100 mM Tris HCl (pH 8.4), 400 mM NaCl, 15 mM MgCl_2_, 2.5mM spermidine), 250μM dNTP mix, 700mM dimethyl sulfoxide (DMSO), 500mM betaine, and 0.2U Taq DNA polymerase (Invitrogen, Cat # M0480S). PCR amplification consisted of a denaturation step at 95 °C for 5 min followed by a two-step touchdown procedure. The first step of 10 cycles consisted of denaturation at 95 °C for 30 s, followed by a primer set-specific annealing for 30 s (annealing temperature decreased by 1 °C per cycle) and elongation at 72 °C for 45 s. The second step of 30 cycles consisted of denaturation at 95 °C for 30 s, followed by annealing (10 °C below annealing temperature used in the first step) for 30 s and elongation at 72 °C for 45 s, followed by a final elongation at 72 °C for 5 min.

The PCR primers for each amplicon were used for bidirectional sequencing using BigDye Terminator Ready Reaction mix according to the manufacturer's instructions. The sequencing products were resolved on an ABI PRISM 3100 DNA analyser (Applied Biosystems), and results were analysed with Applied Biosystems SeqScape software.

### Next-generation whole-exome sequencing

We performed next-generation sequencing to paired-end sequence the exomes of two affected individuals of PKCC009 and PKCC039 at the Cincinnati Children's Hospital and Medical Center (CCHMC) core facility, respectively. Genomic DNAs were captured with the Agilent SureSelect Human All Exon kit according to the manufacturer's instructions (Agilent, Santa Clara, CA). After capture and enrichment, the paired-end libraries were sequenced on the Illumina HiSeq 2000 Genome Analyser (100 bp paired-end reads).

Likewise, the exomes of two affected individuals of PKCC139 were captured, and paired-end sequenced at the EdgeBio Systems (Gaithersburg MD) using Sure Select Human Exome Kit (Agilent Technologies). The paired-end libraries were sequenced on ABI SOLID4 (50 bp paired-end reads). Quality control checks, mapping of the sequenced reads to the reference genome, base calling and subsequent bioinformatic analysis were performed at the above-mentioned facilities.

### Construction of FOXE3 expression plasmids

The wild-type *FOXE3* coding sequence was amplified by PCR and cloned into the entry vector (PCR8/GW/TOPO) according to the manufacturer's instructions (Invitrogen, Foster City, CA). The non-sense (C240*) and two missense (E103K and N117K) alleles of FOXE3 were introduced into the wild-type entry clone (PCR8/GW/TOPO) by site-directed mutagenesis using the QuikChange II XL mutagenesis kit (Cat. # 200521; Agilent, Inc, Santa Clara CA). The coding sequences and orientation of all constructs were confirmed by bidirectional Sanger sequencing. Subsequently, wild-type and mutant FOXE3 entry clones were recombined with pDEST30 and pDEST53 expression vectors using LR Clonase (Cat. #11791-019 Invitrogen, Inc) according to standard gateway protocol following the manufacturer's instructions.

### Cell culture and transfection

HeLa (ATCC; Cat. # CCL-2) and HLE (ATCC; Cat. # CRL-11421) cells were grown on glass coverslips in Dulbecco's Modified Eagle's Medium (DMEM) with 10% foetal bovine serum (FBS) and Eagle's Minimum Essential Medium (EMEM) with 20% FBS, respectively, at 37 °C in a 5% CO2 incubator. About 10 μg plasmid DNA (pDEST53 harbouring either the wild-type *FOXE3* or truncated *FOXE3*) was diluted in 250 μl OptiMEM Reduced Serum Medium (Invitrogen) and incubated for 5 min at room temperature. Parallel to the above incubation, 10 μl of Lipofectamine 2000 diluted in 250 μl of OptiMEM Reduced Serum Medium was incubated for 5 min at room temperature.

Contents of both incubations were mixed well and incubated for an additional 20 min at room temperature. Transfection was performed by adding plasmid-Lipofectamine complex in a six-well plate with a glass cover slip. The cells were then incubated at 37 °C in a 5% CO2 incubator for 24 h.

### Immunofluorescence microscopy

Cells on glass coverslips were washed twice with 1 × PBS (pH 7.4) and then fixed with 6% paraformaldehyde. After fixation, cells were permeabilized with PBS containing Triton X-100 (0.05%), stained with DAPI (4′, 6-diamidino-2-phenylindole; Invitrogen), and images were acquired with a Zeiss microscope.

### Library preparation and next-generation RNA-Seq

For whole-transcriptome sequencing, HEK293FT cells (Cat. #R700-07; Invitrogen) were grown in six-well plates. HEK293FT cells were transfected with the expression vector (pT-Rex-DEST30) harbouring either the wild-type or the mutant (C240*) *FOXE3* open reading frame (ORF) (eight biological replicates for both wild type and mutant), and eight non-transfected biological replicates were used to control the background. Cells were harvested 24 h post transfection and immediately subjected to total RNA isolation for the whole-transcriptome next-generation sequencing libraries. Total RNA quality and quantity were analysed using a NanoDrop Lite spectrophotometer (Thermo Scientific).

Total RNA was used for the preparation of next-generation mRNA sequencing libraries with the TruSeq RNA Sample Preparation Kit v2 (Cat. #RS-122–2001; Illumina) according to the manufacturer's instructions. Briefly, 4 μg total RNA was used for polyA mRNA selection using streptavidin-coated magnetic beads, followed by thermal fragmentation of selected mRNA. The fragmented mRNA was used as a template for cDNA synthesis by reverse transcriptase with random primers. The cDNA was further converted into double stranded DNA that was end-repaired to incorporate the specific index adapters for multiplexing, followed by a purification step and amplification for 15 cycles. The quality and functionality of the final amplified libraries were examined with a DNA high sensitivity chip on an Agilent 2100 Bioanalyzer (Agilent; Palo Alto, CA) and with quantitative PCR (qPCR) according to the manufacturer's instructions. RNA-Seq libraries with unique index sequences were pooled in an equimolar ratio, and a final size selection of 400–500 bp was performed on a Caliper Lab Chip XT DNA 750 chip (Caliper life Sciences, Hopkinton, MA).

Initially, we examined the quality of *FOXE3* transcriptome libraries on a MiSeq genome analyser. After the quality control confirmation, RNA-Seq libraries were sequenced using Illumina TruSeq Cluster V3 flow cell and TruSeq SBS Kit V3 chemistry (Illumina). The 24 mRNA bar-coded pooled libraries were clustered using TruSeq V3 flow cell at a concentration of 13.0 pM and sequenced (2 × 100 bp) in two independent lanes (12 bar-coded mRNA pooled libraries in each lane) on a HiSeq 2000 genome analyser.

Two types of next-generation sequencing data analysis software, a combination of TopHat2 (version 2.0.3.1)/R/Bioconductor package, DESeq and DNASTAR Lasergene Package, a commercially available tool (DNASTAR, Madison, WI), were used for mapping and differential expression studies^35^,^36^. Sequencing reads were subjected to a two-step quality control process for TopHat2 and DESeq analysis. First, the quality of the sequencing reads was evaluated with FastQC. Subsequently, adapter sequences were removed from the reads using SeqPrep (https://github.com/jstjohn/SeqPrep). The processed sequencing reads were aligned against the human reference genome (hg19) as paired-end reads with TopHat2 (version 2.0.3.1) using the default settings^35^. Duplicate reads from PCR amplification were removed, exploiting the rmdup option of samtools^37^.

Differential gene expression analysis was performed using the DESeq R/Bioconductor package with raw read count as input data^36^. The raw read count was calculated using the RPKM_count.py tool in the RSeQC software. DESeq was used to determine significantly up- and downregulated transcripts by a two-step process. In the first step, a cross comparison of the control and the wild-type samples was completed using a FDR of <0.05 that identified a set of transcripts with the 1 × log2-fold change above the background. In the second step, these transcripts were compared with the transcriptome of cells expressing the truncated FOXE3 to identify genes that were differentially expressed in response to the premature termination of FOXE3.

In parallel, RNA-Seq reads (FASTQ) were processed and analysed using DNASTAR. The paired-end reads were assembled with SeqMan NGen version 11, using default parameters. The human genome template package (GRC37.p13) available on the DNASTAR website was used as a reference for mapping RNA-Seq reads. The ArrayStar Version 11 was used for normalization, differential gene expression and statistical analysis of uniquely mapped paired-end reads using the default parameters. The expression data quantification and normalization were calculated using the RPKM value for each transcript gene; the RPKM corresponds to reads per kb per million mapped reads^38^.

Spotfire DecisionSite with Functional Genomics (TIBCO Spotfire, Boston, MA) was used for further evaluation and graphical representation. All transcripts' log2-fold changes between mutant and wild-type samples were analysed to determine the s.d. from their mean of 0, which represents no change. Initially, we chose a 3 s.d.cutoff for differentially expressed genes; however, later we increased the stringency by opting for a 6 s.d. cutoff.

### Sample preparation for multiplex iTRAQ and TMT labelling

HEK293FT cells were transfected with an expression vector (pT-RexTM-DEST30) harbouring either the wild-type or the mutant FOXE3 (C240*) for whole-proteome profiling. Three biological replicates for both the wild-type and mutant FOXE3 were transfected in addition to two non-transfected replicates to estimate the background.

Transfected cells (and non-transfected controls) were washed three times with ice-cold PBS 24 h post transfection and lysed in 0.5% SDS containing 1 mM EDTA and 1 mM phenylmethylsulfonyl fluoride. The cells were lysed using a sonicator (Branson 250 Sonifier) that delivered three 20% pulses each for 5 s on ice. Subsequently, the lysed samples were centrifuged at 15,000*g* for 10 min and protein concentration in the supernatant was measured using the bicinchoninic acid (BCA) method. The protein amounts and reproducibility of protein extraction were confirmed by SDS–PAGE gels. Equal amounts of each protein extract were proteolysed using the FASP (Filter-Aided Sample Preparation) method^39^. Briefly, protein samples were treated with 5 mM TCEP at 37 °C for 45 min followed by 10 mM MMTS for iTRAQ or 10 mM iodoacetamide for TMT (Thermofisher Scientific) at 25 °C for 15 min. Subsequently, 100 μg of each sample was passed three times through a 30 kDa filter (UFC503096, Millipore) using 9 M urea and twice with 50 mM triethylammonium bicarbonate (TEABC). Finally, proteins were proteolysed with Trypsin (V511A, Promega Inc) for 12 h at 37 °C and dried.

The proteolysed peptides from each sample were re-suspended in 37 μl of 250 mM TEABC and labelled with a unique 8PLEX iTRAQ reagent (AB Sciex) according to the manufacturer's instructions. The eight iTRAQ-labelled peptide samples were mixed, dried to remove organic solvents and reconstituted in 10 mM TEABC. Subsequently, the mixture of labelled peptides was fractionated on a RPLC column (XBridge BEH (Ethylene Bridged Hybrid C18 Column, 5 μm, 2.1 × 100 mm via XBridge BEH C18) Guard Column) using an Agilent 1200 HPLC system (Agilent) with a 2% to 90% acetonitrile gradient in TEABC buffer for 80 min (first 40 min linear up to 35% acetonitrile, followed by 20 min linear up to 50% acetonitrile and ended with a 90% acetonitrile concentration in 10 mM TEABC buffer). Peptide fractions from 96 wells were concatenated into 24 fractions, dried and re-suspended in 8 μl of 3% acetonitrile containing 0.1% formic acid for LC-MS/MS analysis.

In parallel, 100 μg of proteolysed protein sample was labelled in 100 μl of 100 mM TEABC with a unique TMT. The labelling reaction was stopped by adding 8 μl of hydroxylamine (5%). The TMT-labelled peptides were mixed, fractionated and concatenated into 24 fractions, dried and re-suspended for LC-MS/MS analysis as described for iTRAQ-labelled peptides.

### Nanoflow electrospray ionization MS/MS

Data-dependent MS/MS analysis of the iTRAQ-labelled peptides was performed on an LTQ Orbitrap Velos (Thermo Scientific) interfaced with Eksigent 2D nanoflow liquid chromatography system (AB Sciex) and an Agilent 1200 micro-well plate auto-sampler. Labelled peptides were trapped and separated on a 2 cm trap column, and a 75 μm × 20 cm nanopore column both packed with Magic C18 AQ, 5 μm, 100 Å (Michrom Bioresources, Auburn Ca) using a 2–90% acetonitrile gradient in 0.1% formic acid over 90 min. Eluting peptides were electrosprayed through a 15-μm emitter (PF3360-75-15-N-5, New Objective) at 2.0 kV spray voltage.

The LTQ Orbitrap Velos was set for full MS survey scan range of 350–1,800 *m/z*, data-dependent HCD MS/MS analysis of top 8 precursors with a minimum signal of 2,000 counts, isolation width of 1.3 Da, 30-s dynamic exclusion limit and normalized collision energy of 40 U. Precursor and the fragment ions were analysed at resolutions of 30,000 and 7,500, respectively. In the case for TMT-labelled peptide samples, resolution for fragmentation ions was set at 30,000 for baseline resolution of TMT reporter ions. Protein ratios were calculated using peptides with <30% co-isolation of co-eluting peptide ions with mass differences <1.3 Da.

### MS data processing protocol

Precursor and fragment ion *m/z* values were extracted from MS raw files using the default spectrum selector, Xtract and MS2-processor nodes in Proteome Discoverer 1.4 software (Thermo Scientific). The extracted data were searched against the RefSeq 2012 human entries using the Mascot (v2.2.6, Matrix Sciences) node in Proteome Discoverer with mass tolerances on precursor and fragment masses set to 15 ppm and 0.03 Da, respectively. Peptide validator node was used for peptide validation, and a 1% FDR cutoff was used to filter the data. Protein ratios were normalized across all samples after excluding peptides with >30% isolation interference from the protein quantification to avoid potential interference of reporter ions from co-isolation of co-eluting peptides with similar masses (<1.3 Da difference).

The search parameters for the iTRAQ data analysis included oxidation of methionine, deamidation on asparagine/glutamine residues, 8PLEX iTRAQ on tyrosine residues and methylthio group on cysteine residues as different variable modifications and iTRAQ-8PLEX on the N terminus and lysine residues as fixed modifications. The search parameters for the TMT data analysis included oxidation of methionine, deamidation of asparagine and glutamine residues as different variable modifications and TMT-8PLEX on the N terminus, lysine residues, and carbamidomethylation of cysteine as fixed modifications.

Proteome data derived from 8PLEX iTRAQ and TMT labelling analysis using the Proteome Discoverer software were imported into Partek Genomics Suite (Partek Inc. Saint Louis MO) for protein annotation and further analysis. Each data set contained three biological replicates representing cells transfected with wild-type and mutant FOXE3 constructs. Both data sets were treated independently to evaluate reproducibility of experiments.

One or more mass spectra are assigned to each NCBI gi identifier, which represents a single protein, and for each protein the median of these multiple spectra values was calculated to produce a single value representing that protein. Only those spectra with a Proteome Discoverer Isolation Interference value of<30% were accepted for further evaluation. The values of each data set's six samples were quantile normalized to minimize the noise and experimental variation and subsequently converted to the log2 notation for statistical analysis.

An analysis of variance using Partek's Student's *t*-test compared each protein's relative concentration between mutant and wild-type cells and determined the statistical significance of that difference regarding its *p*-value. The iTRAQ platform data yielded 3,781 protein comparisons and while the TMT produced 3,315. These values were then exported for further evaluation and graphical representation to Spotfire DecisionSite with Functional Genomics, and all proteins' log2-fold changes between wild-type and mutant FOXE3-expressing samples were analysed to determine the s.d. from their mean of 0, which represents no change. To ensure unambiguous nomenclature and ease of evaluation, all protein gi identifiers were mapped to their cognate genes in the NCBI Entrez database.

### QRT analysis of *Foxe3* and *Dnajb1*

The use of mice in this study was approved by the Johns Hopkins Animal Care and Use Committee (ACUC), and all experiments were performed in accordance with a protocol approved by the Johns Hopkins ACUC. Mouse lenses were obtained at different developmental stages, including embryonic day 12.5 (E12.5), E15, E18, at birth (designated as P0), postnatal day 3 (P3), P6, P9, P12, P14, P21, P28 and P42. Mice were first anesthetized with isoflurane and subsequently killed through cervical dislocation. The ocular tissue was extracted and the lenses were isolated from the retina using forceps under a microscope. The lenses were divided into two pools, each representing biological replicates for the respective developmental stage. Lenses were immediately dissolved in TRIzol reagent (Cat. # 15596-026; Invitrogen; Carlsbad, CA), and total RNA was extracted from each pool according to the manufacturer's instructions. The quality and quantity of the total RNA were determined on a NanoDrop Lite Spectrophotometer.

First-strand cDNA synthesis was completed using the Superscript III kit (Cat. #18080051; Invitrogen) according to the manufacturer's instructions. qRT-PCR was performed on the STEP ONE ABI Real-Time PCR System using pre-designed *Foxe3* and *Dnajb1* TaqMan expression assays (Applied Biosystems). *Gapdh* was used as the endogenous internal control. The Delta–delta *C*_T_ method was used to determine the relative expression, normalized against *Gapdh* expression, at each developmental stage^40^.

### ChIP assay

The Genomatix program (Genomatix, Ann Arbor, MI) was used to identify all transcription factor binding sites within the *DNAJB1* 5′ UTR. Subsequently, a total of eight primer pairs (Supplementary Table 4) were designed to specifically amplify the target regions of the two *DNAJB1* isoforms. HLE cells were seeded in 6-well plates. The ChIP experiment was performed using a ChIP-IT Express Enzymatic Magnetic Chromatin immunoprecipitation kit (Cat. # 53009 and 53035; Active Motif, CA). Chromatin of the cells was then cross-linked with 1% formaldehyde for 10 min and subsequently quenched with 125 mM glycine for 5 min. Cells were washed with cold PBS and nuclei extracted with cell lysis buffer. Cross-linked chromatin was sheared with enzymatic shearing by incubation with a working stock of Enzymatic Shearing Cocktail (200 μl ml^−1^) at 37 °C for 5 min, yielding products of 200–1,000 bp in length. The reaction was terminated by adding EDTA, followed by centrifuging the sheared chromatin in the supernatant as the ‘input DNA.'

The IP reactions were set up by adding ChIP buffer, protein G magnetic beads, protease inhibitor cocktail, sheared chromatin and anti-FOXE3 antibody (Cat. # SC-48162; Santa Cruz Biotechnology, Santa Cruz, CA) overnight with rotation at 4 °C. Immuno-complexes were then captured with protein G beads. Complexes were washed and eluted with elution buffer. Both eluted and input DNA were treated with proteinase K (215 mg ml^−1^) and incubated at 37 °C for 1 h. Finally, the tubes were returned to room temperature and 2 μl of proteinase K stop solution was added. The pull-down DNA was then analysed using *DNAJB1* primers pairs to determine whether the 5′ UTR was a part of the nucleoprotein complex with FOXE3.

### Luciferase reporter assay

DNAJB1, a transcriptional target of FOXE3, was further evaluated by luciferase reporter assays (GeneCopoeia, Rockville, MD) in HEK293FT cells. A promoter-less or negative control vector (Cat. #NEG-PG04), a positive control vector (Cat. #GAPDH-PG04) and two custom designed GLuc-SEAP vectors (termed P1 and P2 harbouring 772 and 1,521 bp of putative FOXE3-binding sites described above for ChIP assay) were acquired commercially (GeneCopoeia, Gaithersburg MD). HEK293FT cells were seeded in a six-well plate and co-transfected with *DNAJB1*-GLuc-SEAP vectors (P1 or P2) and a plasmid expressing either the wild-type or mutant FOXE3 alleles (E103K, N117K and C240*).

The transfection of *DNAJB1*-GLuc-SEAP vectors (P1 and P2) alone served as controls to measure the background luciferase activity. Growth media was collected 24 h post transfection, and the Gaussia luciferase (Gluc) activities were measured using a Secrete Pair Dual Luminescence assay kit (Cat. # SPDA-D010) according to the manufacturer's instructions (GeneCopoeia). The alkaline phosphatase (SEAP) reporter, driven by the CMV promoter, cloned into the luciferase vector served as an internal control to normalize for transfection efficiency.

### Examining the effect of the mutant alleles on *DNAJB1* expression

HEK293FT cells were transfected with the expression plasmid (pT-Rex-DEST30) harbouring either the wild-type *FOXE3* or the mutant alleles (E103K, N117K and C240*). HEK293FT cells were seeded in a six-well plate and transfected in triplicates with plasmids harbouring the wild-type or mutant *FOXE3* alleles. Non-transfected HEK293FT cells and HEK293FT cells transfected with empty pT-Rex-DEST30 served as controls to normalize the background.

Total RNA was isolated using TRIzol reagent according to the manufacturer's instructions (Invitrogen), and RNA concentrations were determined using a NanoDrop Lite Spectrophotometer. The first-strand cDNA synthesis was carried out using Superscript III kit according to the manufacturer's instructions. Pre-designed human *DNAJB1* and *GAPDH* TaqMan probes and primers were used for qRT-PCR. The Delta–delta *C*_T_ method was used to determine the expression of *DNAJB1* normalized against *GAPDH*^40^.

### ShRNA-guided knockdown of *DNAJB1* in HLE cells

A cocktail of four pre-designed shRNA in a lentiviral GFP vector targeting *DNAJB1* was purchased from Origene (Cat #TL313427) along with scrambled shRNA (Cat #TR30021). HLE cells were grown in T-75 flasks using EMEM supplemented with 20% FBS, 100 U ml^−1^ penicillin and 100 μg ml^−1^ streptomycin at 37 °C in a 5% CO_2_ incubator. Cell density (confluence) of HLE cells was determined using an automated cell counter (Countess; Invitrogen). Approximately 6 × 10^6^ cells were pelleted (100*g*) and re-suspended in a cocktail of SE solution and supplement according to the manufacturer's instructions (Lonza, Germany), and 20 μl aliquots were used for each transfection.

Transfections were performed in triplicate for both *DNAJB1* and scrambled shRNAs (used as a control) in a 16-well nueleocuvette strip (Lonza 4D-Nucleofector) using 1 μg of plasmid (250 ng each of the four shRNAs targeting *DNAJB1* or 1 μg of the scrambled shRNA). After electroporation, cells were immediately re-suspended in EMEM growth medium and were gently transferred to a 100 mm cell culture petri dish. The growth media of transfected cells was changed 6 h after the transfection and cells were harvested 48 h post transfection. The harvested cells were subjected to total RNA isolation, cDNA synthesis and *DNAJB1* expression quantification using the TaqMan assay as described.

The TUNEL assay was used to investigate the effect of *DNAJB1* knockdown in HLE cells. A total of three independent transfections in duplicates were performed for both *DNAJB1* and scrambled shRNA plasmids as described. Cells were harvested 48 h post transfection and washed with ice-cold PBS. These cells were fixed with 1% PFA on ice for 20 min and subsequently processed for TUNEL staining using an Apoptag Red *In Situ* Apoptosis Detection Kit (Cat. #S7165, EMD Millipore) according to the manufacturer's instructions. Approximately 30,000 cells per sample were collected using an LSR II Flow Cytometer (Becton Dickinson, Bedford, MA) and apoptosis positive and negative cells were determined using FlowJo Version 9.5 (Tree Star, Inc Ashland, OR).

The effect of *DNAJB1* knockdown on HLE cell cycle was examined through flow cytometry of *DNAJB1* shRNA-transfected cells. A total of three independent transfections in triplicate were performed for both *DNAJB1* and scrambled shRNA plasmids as described. HLE cells were harvested 48 h post transfection after washing two times with ice-cold PBS and fixed with 1% PFA for 10 min on ice. After fixation, HLE cells were washed two times with ice-cold PBS, stained with PI solution (0.1% Triton X-100, 10 μg ml^−1^ RNase A and 20 μg ml^−1^ PI stock solution in PBS) and stored, protected from light, at 4 °C until analysis. Approximately 30,000 cells per sample were collected using a FACSCalibur (Becton Dickinson). The cell cycle stage was determined using FlowJo Version 9.5. Whole cells were identified using a Forward versus Side Scatter gate. Single cells were detected using an FL2-Area versus FL2-Width gate. Watson (Pragmatic) modelling was used to estimate the percentage of the cell population at the G0/G1, S and G2/M phases and the Sub G0/G1 population.

### Investigating the effects of *dnajb1* knockdown *in vivo*

sbMOs against *dnajb1a* (5′-CCTTTGAGCCCTGAGGAGAAATAAA-3′), targeting the intron 1/exon 2 junction (Supplementary Fig. 8a) and *dnajb1b* (5′-GCATATTTTTACTCACCTTCTTCGC-3′), targeting the exon 1/intron 1 junction (Supplementary Fig. 9a) were designed and synthesized by Gene Tools, LLC (Philomath, OR). In addition, a tbMO against *dnajb1b* (5′-TCTTTCCCCATCTTCACCATTAGGC-3′) was synthesized by Gene Tools, LLC. The use of zebrafish in this study was approved by the Johns Hopkins ACUC, and all experiments were performed in accordance with a protocol approved by the Johns Hopkins ACUC.

The morpholinos were injected into one- to two-cell stage zebrafish embryos. MO efficacy and optimal concentration were determined by a dose–response curve ranging from 1 to 10 ng. Control embryos were injected with 5 ng of a non-specific control MO (Gene Tools, LLC). The efficacy of the sbMO was examined through qRT-PCR. Approximately, 15–20 embryos were collected at 12 and 24 h post- injection and total RNA was extracted using TRIzol reagent. First-strand cDNA synthesis was completed using Superscript III according to the manufacturer's instructions. Three primers annealing to exon 1 (forward; 5′-CGGAGCAGAGGATAAATTCAAAG-3′), exon 2 (reverse; 5′-AAAGTGTAGCTAGGGCCATTG-3′) and exon 3 (reverse 5′-CCTTCTCCCACAATCCTCTTC-3′) were used to quantitate *dnajb1a* in morphant and control cDNAs. Likewise, primer pairs annealing to exon 1 (forward; 5′-AGCCGAGGAAAAGTTCAAGG-3′) and exon 2 (reverse; 5′-GAGCACCTCCCTTCAGTC-3′) were used to quantitate *dnajb1b* in morphant and control cDNAs. *actb* (forward; 5′-GCCAACAGAGAGAAGATGACACAG-3′ and reverse; 5′-CAGGAAGGAAGGCTGGAAGAG-3′) was used as an internal control to normalize the expression of *dnajb1a* and *dnajb1b*. The qRT-PCR was performed using Power SYBR Green PCR Master Mix (Cat. # 4367659; Life Technologies, CA) according to the manufacturer's instructions. The relative gene expression was calculated with the delta–delta *C*_T_ method for *dnajb1a* and *dnajb1b* in morphant and control cDNAs.

Embryos were raised until 4 dpf, immobilized by the addition of Tricaine (Cat. # MS-222, Sigma) to culture media, and imaged live by bright field microscopy to determine if general developmental or morphological defects were present. *dnajb1a* mRNA was transcribed from the pCS2+ expression vector containing the full-length wild-type ORF of the gene. For rescue experiments, 150 pg mRNA encoding full-length wild-type *dnajb1a* was co-injected along with the MO. Approximately 100 embryos were injected and scored per treatment for either MO alone or MO with *dnajb1a* mRNA. The rescue efficiency of the zebrafish *dnajb1a* MO was measured by scoring the proportion of embryos exhibiting normal eye morphology. Embryos were scored blind to the injection cocktail. In parallel, zebrafish embryos at 4 dpf were anesthetized in tricaine and fixed in 4% paraformaldehyde overnight, embedded in paraffin and sectioned at 5-μm thickness for haematoxylin and eosin and TUNEL staining.

Paraffin-embedded sections of zebrafish embryos (4 dpf) were deparaffinized and rehydrated in PBS. The sections were treated with 10 μg ml^−1^ of Proteinase K in PBS and washed three times with PBS. An Apoptag Fluorescein *in situ* detection kit (Cat. #S7110, EMD Millipore) was used for TUNEL staining according to the manufacturer's protocol for tissue sections. The sections were mounted with Prolong antifade mounting medium with DAPI (Life Technologies) and imaged on a Zeiss Axioskope microscope using ImagePro software.

## Additional information

**Accession codes:** All raw and processed RNA-Seq data reported in this manuscript have been deposited in NCBI's Gene Expression Omnibus (GEO), and are accessible through GEO series accession number GSE76389. The mass spectrometry proteomics data have been deposited to the ProteomeXchange Consortium via the PRIDE partner repository with the data set identifier PXD003436.

**How to cite this article:** Khan, S. Y. *et al*. *FOXE3* contributes to Peters anomaly through transcriptional regulation of an autophagy-associated protein termed DNAJB1. *Nat. Commun.* 7:10953 doi: 10.1038/ncomms10953 (2016).

## Supplementary Material

Supplementary Information Supplementary Figures 1-10 and Supplementary Tables 1-2

Supplementary Data 1 The list of unique peptides and spectra used for proteins quantification identified in HEK293FT cells expressing the wild type and mutant FOXE3 through the iTRAQ proteome profiling experiment.

Supplementary Data 2 The list of unique peptides and spectra used for proteins quantification identified in HEK293FT cells expressing the wild type and mutant FOXE3 through the TMT proteome profiling experiment.

Supplementary Data 3 The list of primers used to sequence FOXE3

Supplementary Data 4 The list of primers used in the ChIP analysis

## Figures and Tables

**Table 1:** A summary of the transcriptome data of control, wild-type and truncated FOXE3-expressing cells.

Sample	Sample ID	Total reads (10^6^)	Total mapped reads (10^6^)	Total mapped reads w/o PCR duplication (10^6^)	% Total mapped reads w/o PCR duplication	Properly paired reads (10^6^)	% properly paired reads	Sequenced bases (Mb)	∼Human transcriptome coverage (×)
*Lane-1: whole-transcriptome sequencing (2 × 100 bp)*									
Control	CNT-A1	29.62	25.50	24.50	87.72	19.32	78.87	1,932.76	29.73
	CNT-A2	34.17	29.52	28.62	83.75	22.94	80.17	2,294.60	35.30
	CNT-A3	29.39	25.33	24.60	83.72	19.93	81.00	1,993.18	30.66
	CNT-A4	30.13	26.08	25.50	84.63	20.71	81.21	2,071.26	31.86
*FOXE3* mutant	MUT-A1	26.35	21.89	21.06	79.92	16.31	77.45	1,631.06	25.09
	MUT-A2	24.93	20.78	20.30	81.42	16.30	80.30	1,630.05	25.07
	MUT-A3	23.76	19.70	19.27	81.10	15.45	80.19	1,545.44	23.77
	MUT-A4	29.95	25.01	24.43	81.58	19.58	80.15	1,958.61	30.13
*FOXE3*-wild type	WT-A1	28.01	23.30	22.48	80.28	17.49	77.81	1,749.62	26.91
	WT-A2	29.25	24.91	24.27	82.99	19.64	80.93	1,964.89	30.22
	WT-A3	31.18	26.36	25.72	82.49	20.87	81.16	2,087.69	32.11
	WT-A4	31.35	26.52	25.81	82.33	20.94	81.16	2,094.79	32.22
Total		348.13	294.95	286.61	82.24	229.53	80.09	22,953.99	353.13
									
*Lane-2: whole-transcriptome sequencing (2 × 100 bp)*									
Control	CNT-B1	31.24	26.66	25.42	81.37	19.33	76.03	1,933.31	29.74
	CNT-B2	27.57	23.58	22.95	83.23	17.86	77.86	1,786.92	27.49
	CNT-B3	28.73	24.82	24.19	84.20	19.13	79.09	1,913.35	29.43
	CNT-B4	28.91	24.90	24.24	83.84	19.10	78.79	1,910.40	29.39
*FOXE3* mutant	MUT-B1	30.74	25.30	24.51	79.73	18.64	76.07	1,864.83	28.68
	MUT-B2	30.13	25.02	24.62	81.71	19.34	78.57	1,934.69	29.76
	MUT-B3	32.35	27.17	26.46	81.79	20.79	78.59	2,079.85	31.99
	MUT-B4	31.89	26.74	26.14	81.97	20.59	78.80	2,059.92	31.69
*FOXE3*-wild type	WT-B1	26.06	21.70	20.98	80.53	15.98	76.15	1,598.17	24.58
	WT-B2	34.50	29.08	28.43	82.40	22.51	79.21	2,251.94	34.64
	WT-B3	29.99	25.51	24.94	83.16	19.85	79.62	1,985.69	30.54
	WT-B4	32.18	27.25	26.69	82.93	21.37	80.08	2,137.50	32.88
Total		364.35	307.79	299.91	82.23	234.56	78.24	23,456.61	360.87
Combined (lanes 1 and 2)		712.48	602.75	586.52	82.24	464.61	79.14	46,410.61	714.00

CNT, control; MUT, mutant; WT, wild type.
