# Supplementary material for: FOXE3 contributes to Peters anomaly through transcriptional regulation of an autophagy-associated protein termed DNAJB1
Source: Nat Commun. 2016 Mar 24;7:10953. doi: 10.1038/ncomms10953 (PMC4820811; doi:10.1038/ncomms10953)
Supplement: Supplementary Information — Supplementary Figures 1-10 and Supplementary Tables 1-2 [file ncomms10953-s1.pdf]

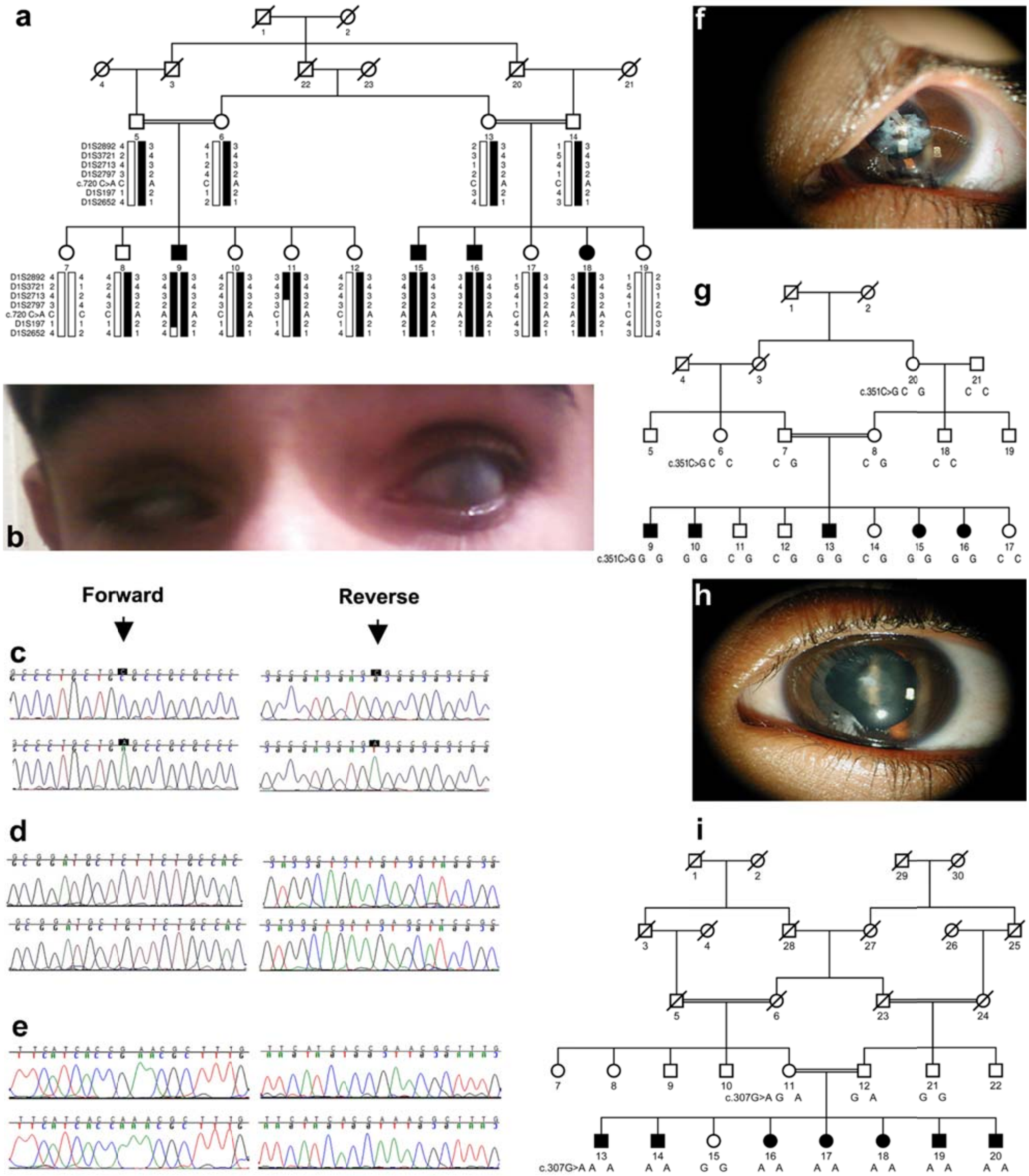

Supplementary Figure 1: Illustration of the disease phenotype and the genetic determinants of families PKCC139, PKCC009, and PKCC039 harboring mutations in *FOXE3*. a) Pedigree drawing of PKCC139 with haplotypes of chromosome 1p STR (short tandem repeat) markers. Squares: males; circles: females; filled symbols: affected individuals; double line between individuals: consanguinity; and diagonal

line through a symbol: deceased family member. Haplotypes of six adjacent chromosome 1p microsatellite markers are shown with alleles forming the risk haplotype shaded black and alleles not co-segregating with cataracts are shown in white. b) Affected individual 9 of PKCC139 exhibiting corneal opacity. c) Forward and reverse sequence chromatograms of individuals 7 (unaffected) harboring the wild type allele and individual 9 (affected) homozygous for a single base change: c.720C>A (p.C240*) in PKCC139. Note: The truncation mutation segregates with the disease phenotype (Peters anomaly) in PKCC139.d) Forward and reverse sequence chromatograms of individual 17 (unaffected) homozygous for the wild type allele and individual 13 homozygous for the c.351C>G (p.N117K) substitution in PKCC009. c) Forward and reverse sequence chromatograms of individual 10 homozygous for the wild type allele and individual 11 homozygous for c.307G>A, (p. E103K) substitution in PKCC039. Note: arrows point to base-pair substitutions in *FOXE3* present in PKCC139, PKCC009, and PKCC039. Forward and reverse sequence chromatograms of d) individual 17 (unaffected) homozygous for the wild type allele and individual 13 homozygous for the c.351C>G (p.N117K) substitution in PKCC009, and e) individual 10 homozygous for the wild type allele and individual 11 homozygous for c.307G>A, (p. E103K) substitution in PKCC039. Note: arrows point to base-pair substitutions in *FOXE3* present in PKCC139, PKCC009, and PKCC039. f) Image of bilateral membranous cataracts present in individual 9 of PKCC009. g) Pedigree illustrating segregation of c.351C>G (p.N117K) with non-syndromic congenital cataracts in PKCC009. h) Image of bilateral posterior sub capsular cataracts present in affected individual 19 of PKCC039. i) Pedigree illustrating the segregation of c.307G>A (p.E103K) with non-syndromic congenital cataracts in PKCC039.

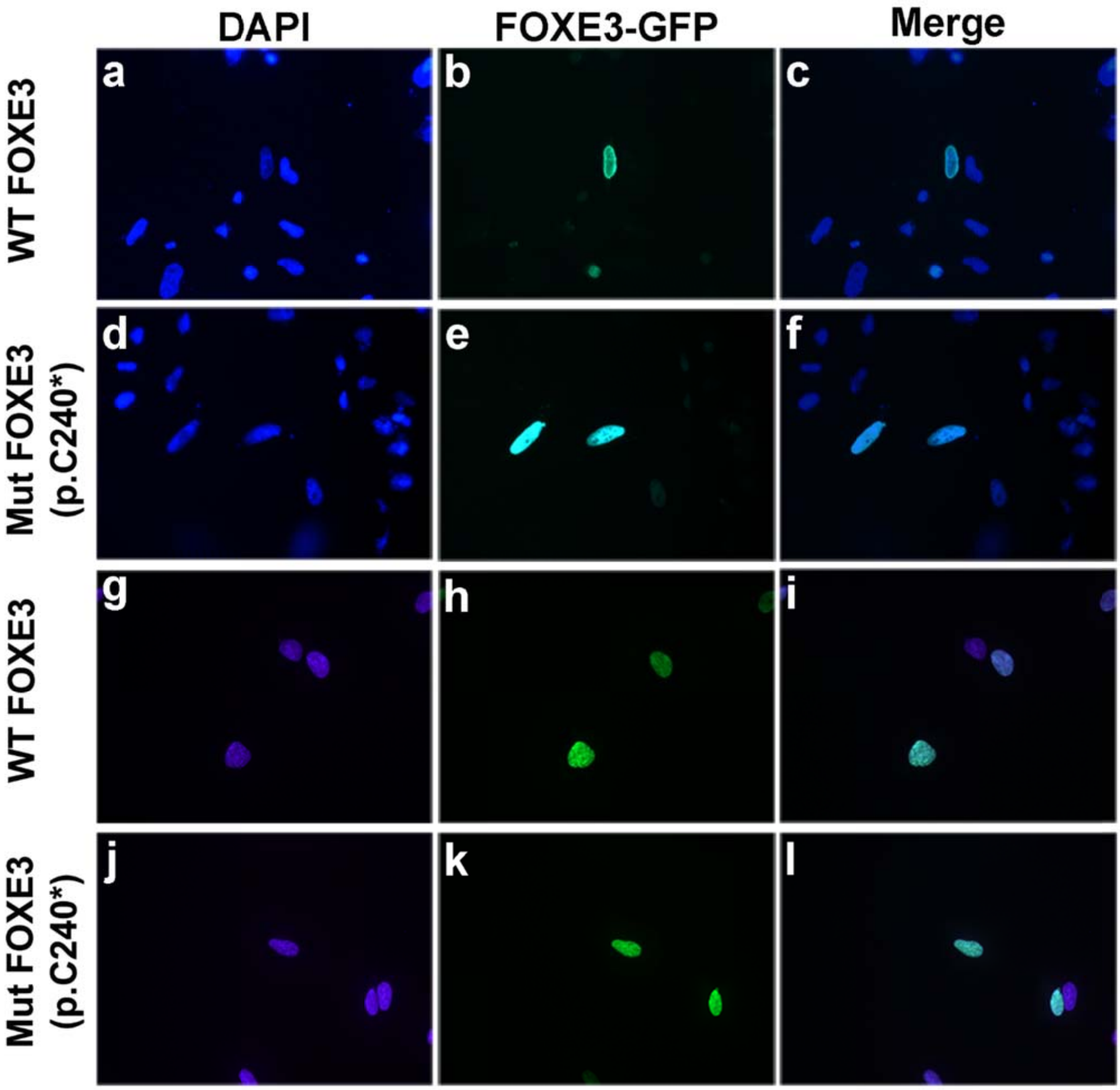

Supplementary Figure 2: Sub-cellular localization of wild-type and mutant *FOXE3* in HeLa and human lens epithelial (HLE) cells. Both HeLa (a-f) and HLE (g-l) cells were transfected with wild-type and mutant (p.C240*) *FOXE3* constructs (pDEST53-Invitrogen) fused in frame with N-terminus GFP. a, d, g, j) nuclear staining with DAPI; b, h) wild type and e, k) mutant FOXE3-GFP flourscence; and c, f, i, l) merge of DAPI and GFP flourscence, respectively. Panels c and i (Wild type) and f and l (mutant) illustrate an indistinguishable pattern of localization to the nucleus in both HeLa and HLE cells.

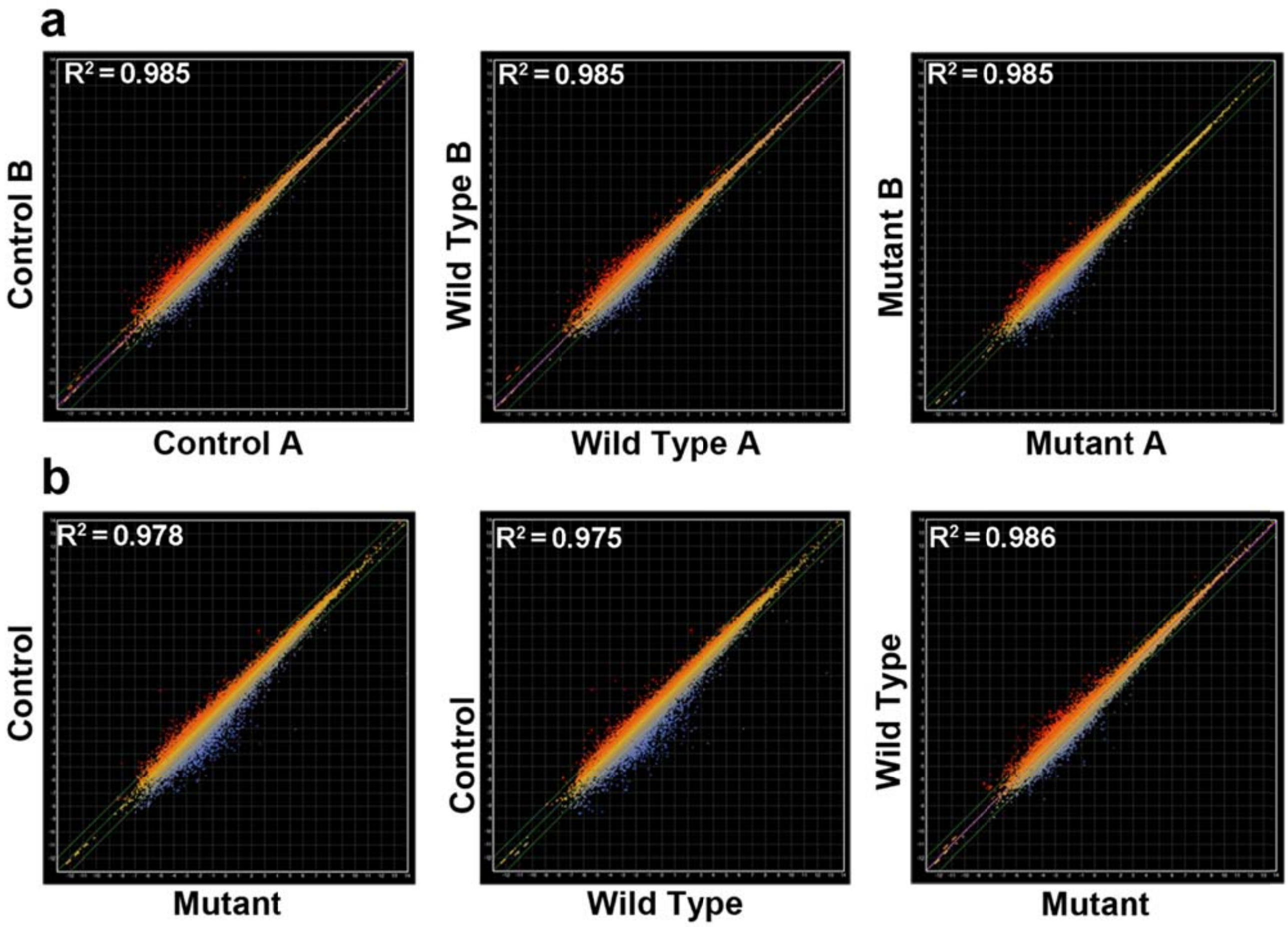

Supplementary Figure 3: Scatter plots of the transcriptome analysis showing correlation among different biological replicates within each experimental condition. Eight biological replicates for each experimental condition (i.e. wild type, mutant, and control) were sequenced in two HiSeq2000 lanes (designated as a & b). The $\log_2$ (logarithmic base 2) scale was used to present the gene expression of each biological replicate in the scatter plots. Scatter plots showing correlation of biological replicates A and B for each experimental condition revealed a high Pearson correlation (R^2) value of 0.985 for each biological replicate. Pearson correlation (R^2) values of 0.978 and 0.975 were obtained from comparisons of the gene expression between control and mutant samples and control and wild type samples, respectively. Comparison of the expression in the wild type and the mutant samples resulted in an R^2 value of 0.986.

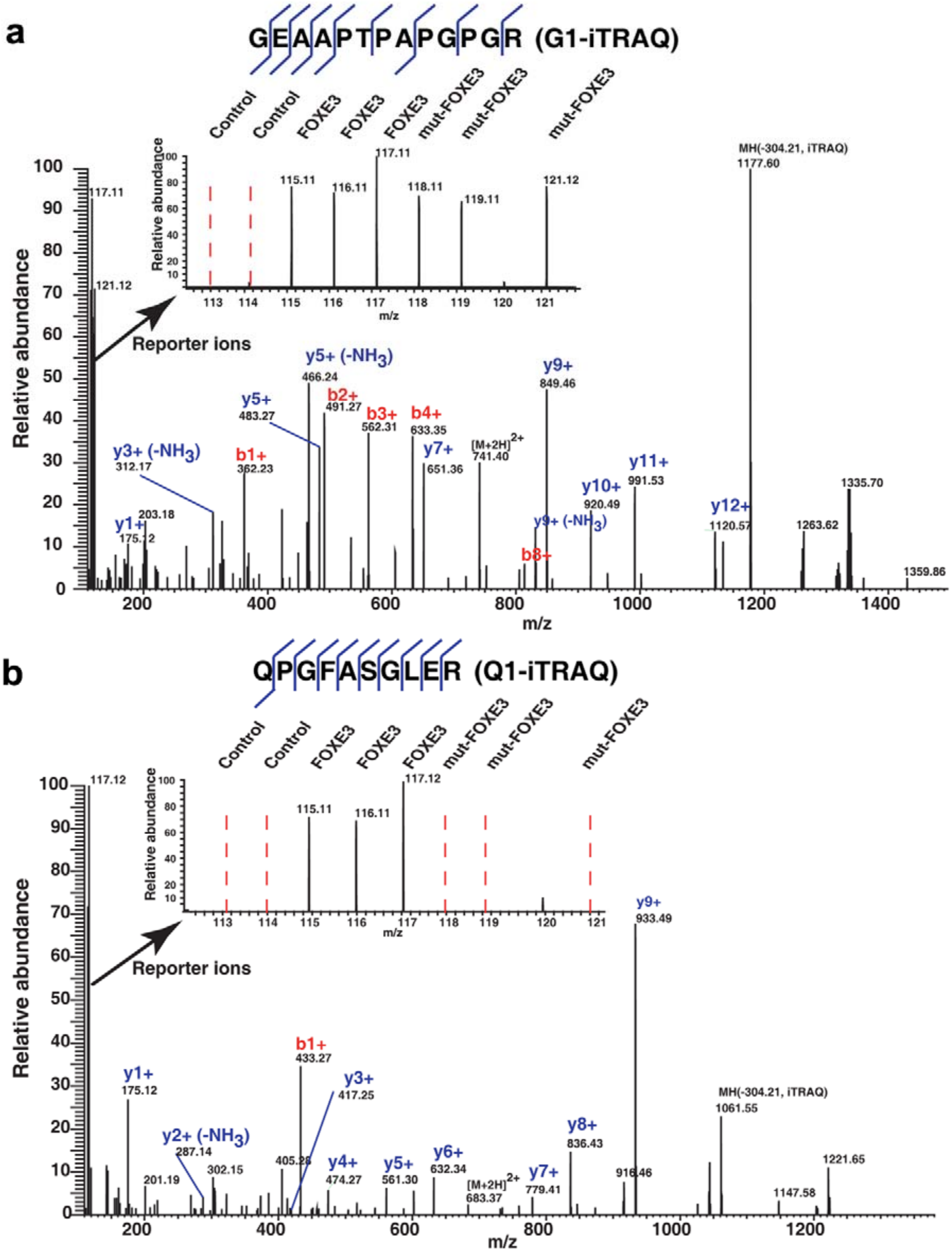

Supplementary Figure 4: Quantitative level of wild type and the p.C240* mutant FOXE3 in HEK293FT cells. Annotated MS/MS spectra of peptides from a) N-terminal region GEAAPTPAPGPGR and b) C-terminal region QPGFASGLER of FOXE3 are illustrated. Reporter ion region (arrow) from N-terminal

region peptide (a) shows equal expression in wild type (reporter ions 115, 116 and 117) and mutant (reporter ions 118, 119 and 121) cell lines and absence (dotted lines) in control cells (reporter ions 113 and 114), whereas reporter ion spectra from C-terminal region peptide (b) show complete absence (dotted lines) of wild type FOXE3 in mutant and control cells.

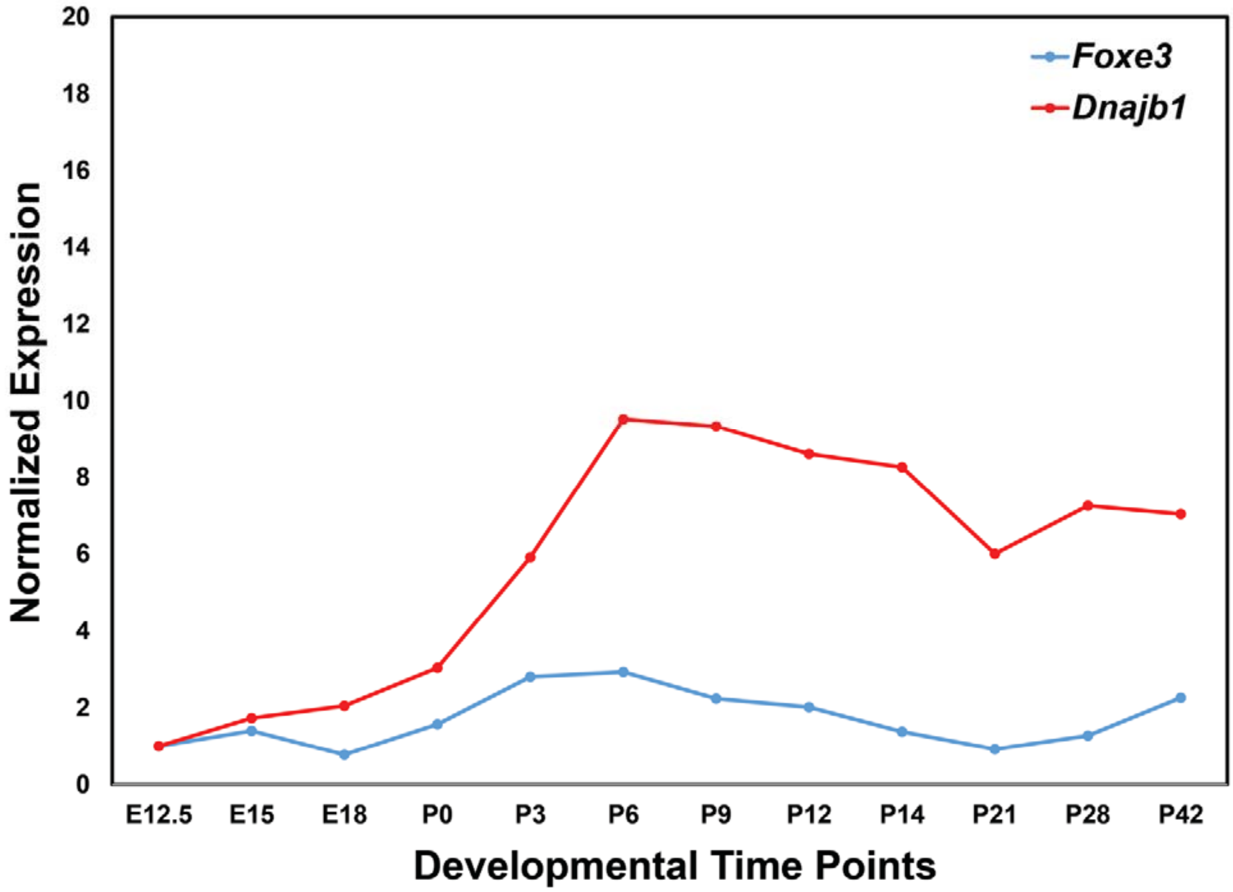

Supplementary Figure 5: Relative expression of *Foxe3* and *Dnajb1* in developing mouse lenses examined at multiple time points. The expression was normalized with *Gapdh*, and the embryonic day 12.5 (E12.5) was chosen as a reference point. The x-axis and y-axis represent time points and normalized expression, respectively.

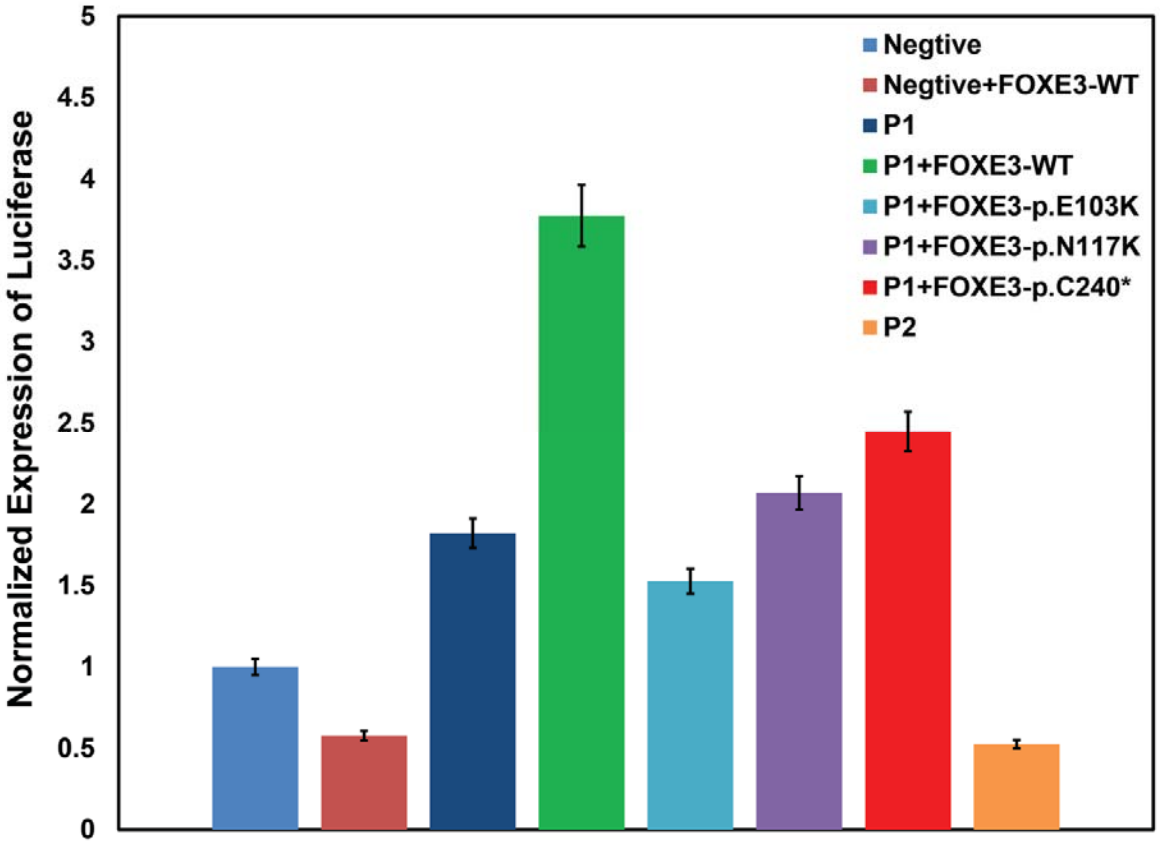

Supplementary Figure 6: Luciferase reporter assay illustrating the compromised transcriptional activities of mutant FOXE3 proteins. The DNAJB1-GLuc-SEAP vectors (designated as P1 and P2) were transfected with expression vectors harboring either the wild-type or the mutant *FOXE3* (p.E103K, p.N117K and p.C240*) alleles in HEK293FT cells normalized with a promoter-less or negative control vector. Error bars represent the standard deviation of three replicates luciferase activity normalized to SEAP.

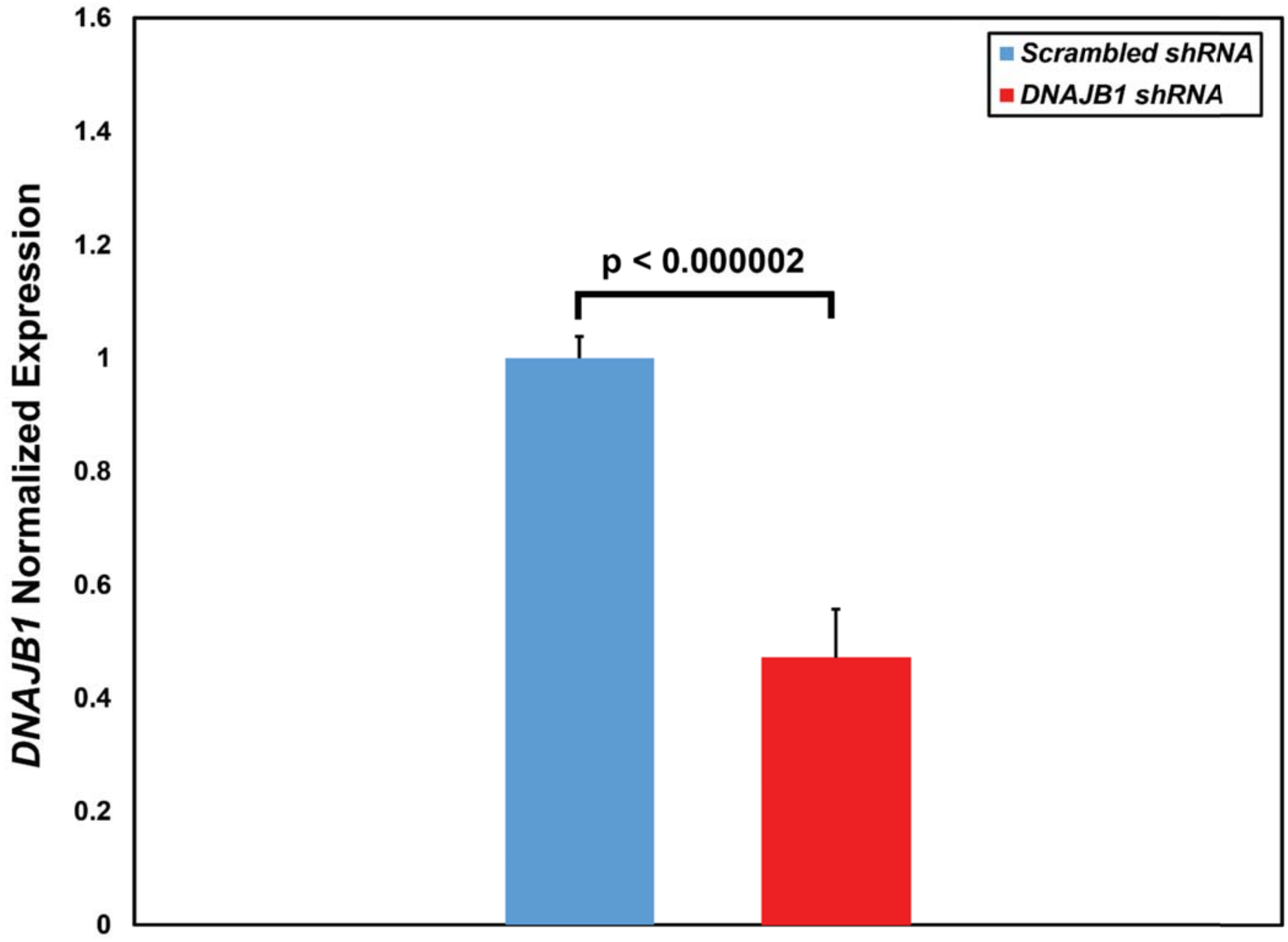

Supplementary Figure 7: Knockdown of *DNAJB1* expression using shRNA in HLE cells. A cocktail of four pre-designed shRNAs was used to knockdown *DNAJB1* expression while a scrambled shRNA served as control. Quantitative real-time PCR analysis revealed a 50% reduction in expression of *DNAJB1* when compared to the expression of *DNAJB1* cells transfected with a scrambled shRNA. Two separate transfections were performed for both *DNAJB1* and scrambled shRNA in duplicates. Error bars represent the standard deviation of four replicates ($p<0.000002$; 2-tailed student's t-test).

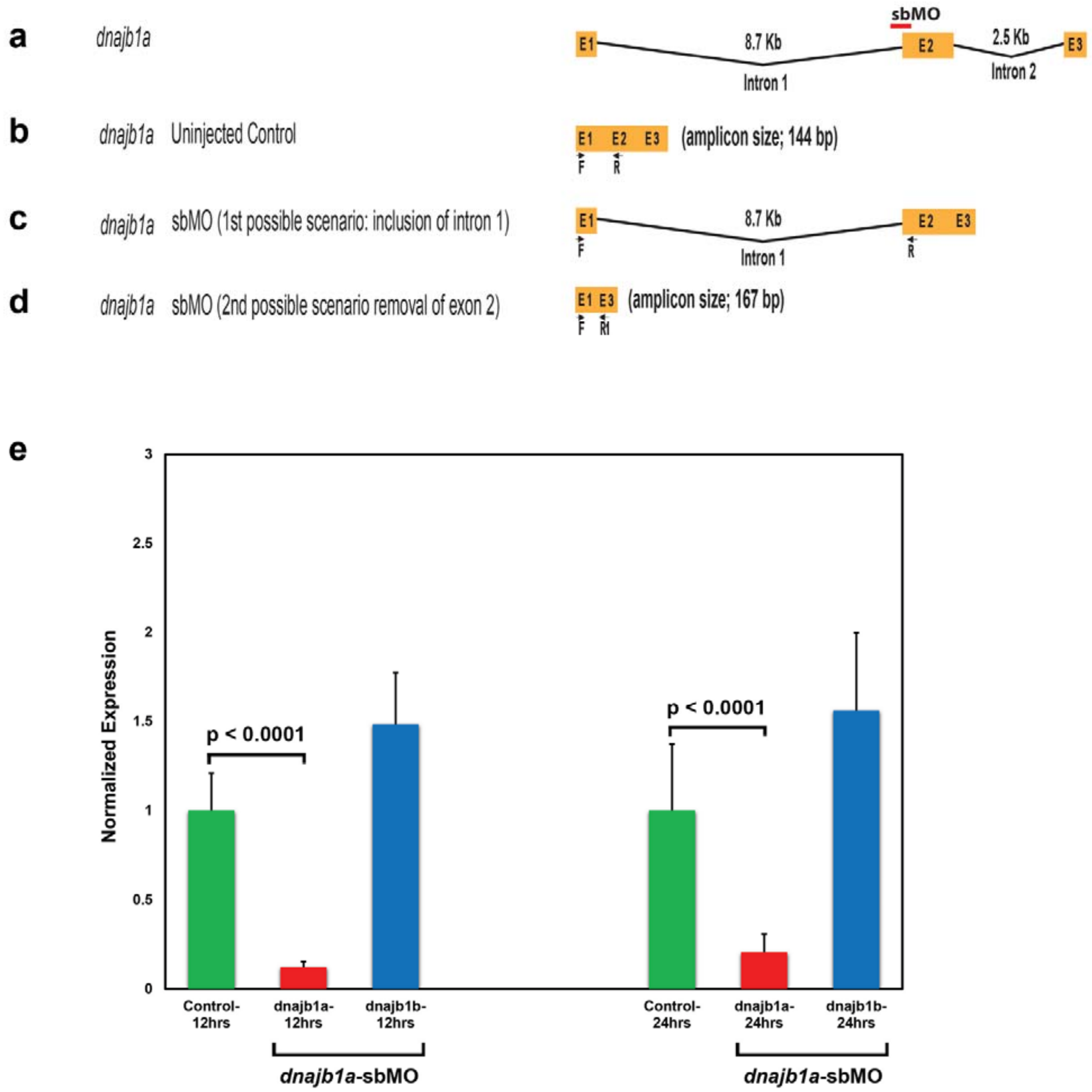

Supplementary Figure 8: Evaluation of the efficacy of splicing-site blocking morpholinos (sbMO) targeted against *dnajb1a* through RT-PCR. a) Schematic illustration of a morpholino-based knockdown of *dnajb1a* mRNA splicing. The red bar represents the binding site for sbMO (splicing-site blocking morphilino). b) mRNA splicing removes introns 1 and 2 that result in mature spliced *dnajb1a* mRNA. F and R represent forward and reverse primers that would yield 144 bp PCR product when the wild type spliced *dnajb1a* mRNA is the template. The sbMO is expected to inhibit the normal splicing of *dnajb1a* resulting in either c) retaining intron 1 or d) excising exon 2 along with introns 1 and 2. e) 2ng of the *dnajb1a* sbMO

were injected at 1-2 cell stage zebrafish embryos. A total of 15-20 embryos were collected at 12 and 24 hours post-injection, and total RNA was extracted to prepare cDNA as described in materials and methods. A primer pair annealing to exons 1 and 2 was used to quantitate *dnajb1a* in morphant and control cDNAs. Only a small fraction of wild type splicing product was detected in *dnajb1a* morphant cDNA both at 12 and 24 hours time-points confirming the efficacy of the dnajb1a sbMO against its respective target. Error bars represent the standard deviation in each dataset of *dnajb1b* and *dnajb1a* expression normalized against β-actin analyzed in six independent experiments ($p<0.0001$; 2-tailed student's t-test).

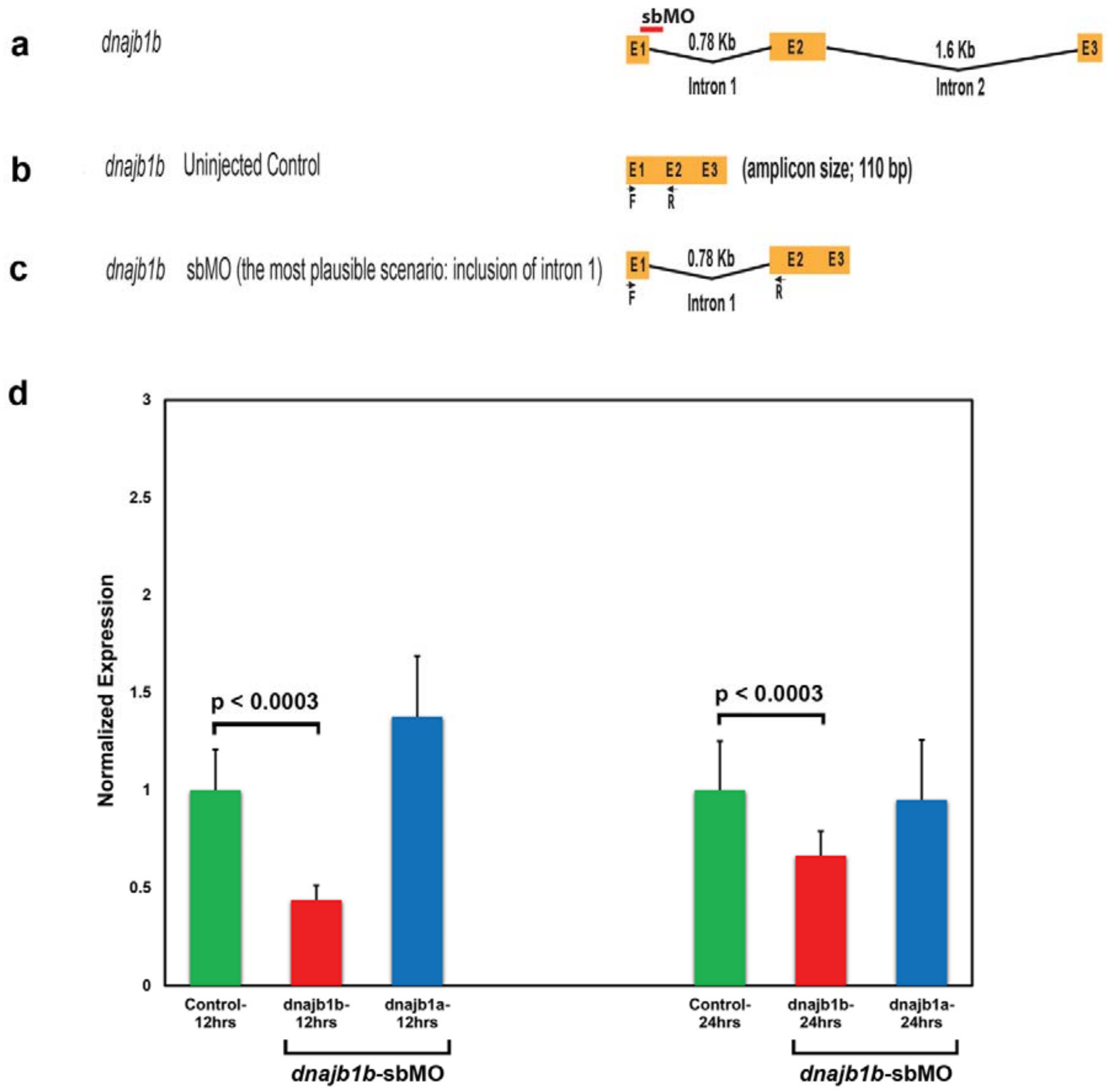

Supplementary Figure 9: Evaluation of the efficacy of splicing-site blocking morpholinos (sbMO) targeted against *dnajb1b* through RT-PCR. a) Schematic illustration of a morpholino-based knockdown of *dnajb1b* mRNA splicing. The red bar represents the binding site for sbMO (splicing-site blocking morphilino). b) mRNA splicing removes introns 1 and 2 that result in mature spliced *dnajb1b* mRNA. F and R represent forward and reverse primers that would yield a 110 bp PCR product when the wild type spliced *dnajb1b* mRNA is the template. c) The sbMO is expected to inhibit normal splicing of *dnajb1b* resulting in retention of intron 1. d) 10ng of *dnajb1b* was injected at 1-2 cell stage zebrafish embryos. A total of 15-20 embryos were collected at 12 and 24 hours post-injection, as for *dnajb1b* above. A primer pair annealing to exons 1 and 2 was used to quantitate *dnajb1b* in morphant and control cDNAs. Only a small fraction of wild type splicing product was detected in *dnajb1b* morphant cDNA both at 12 and 24 hour time-points

confirming the efficacy of *dnajb1b* sbMO against its respective target. Error bars represent the standard deviation in each dataset of *dnajb1b* and *dnajb1a* expression normalized against β-actin analyzed in six independent experiments ($p<0.0003$; 2-tailed student's t-test).

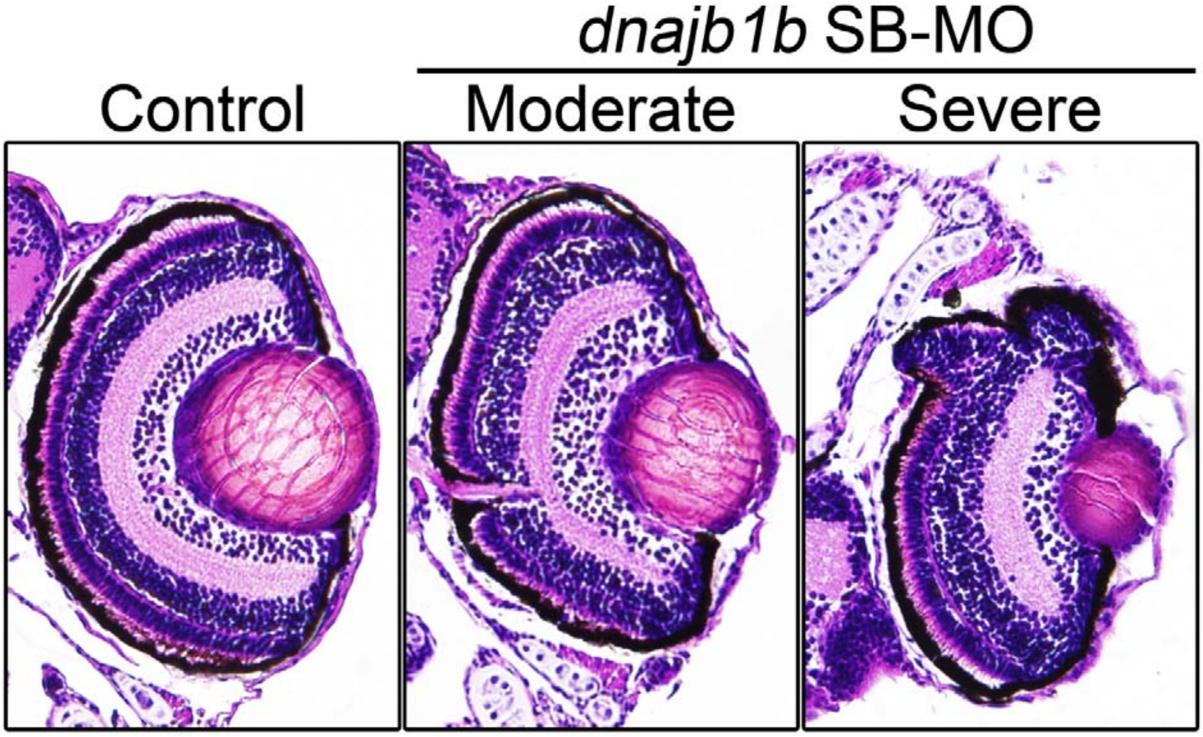

Supplementary Figure 10: Histological evaluation of the developmental defects in the anterior segment of *dnajb1b* morphant eyes. Zebrafish embryos were injected with a morpholino (10ng), and 4-days post fertilization embryos were fixed in 4% paraformaldehyde and embedded in paraffin. 5µm sections were stained with hematoxylin and eosin. The less severe morphant embryos showed protruding lenses while the severely affected morphant embryos demonstrated smaller eyes with somewhat elongated/ bulged lenses compared to the controls.

Gene	CONTROL - linear	WILD TYPE - linear	MUTANT - linear	Fold change	p value
**Down Regulated in Mutant *FOXE3***					
*DNAJB1**	74.027	797.521	217.585	0.273	0.000
*DUSP10**	2.781	7.676	4.251	0.554	0.001
*PCDH18**	3.773	9.434	2.530	0.268	0.002
*COL3A1**	1.075	3.222	0.965	0.300	0.002
*FILIP1L**	0.245	5.796	1.876	0.324	0.004
HSP90AA4P	40.665	61.463	40.754	0.663	0.004
*HSPA1L**	4.832	18.327	7.976	0.435	0.004
*ARID5B**	3.898	12.349	7.793	0.631	0.006
*DEDD2**	6.704	29.330	9.485	0.323	0.008
EPS8L2	0.409	3.514	1.975	0.562	0.008
ACTRT3	4.569	9.664	4.967	0.514	0.010
*GRHL1**	5.993	9.826	6.511	0.663	0.010
*PRKACB**	1.324	4.324	0.714	0.165	0.011
LOC100130548	0.436	3.831	0.752	0.196	0.012
C6orf165	0.688	2.930	1.442	0.492	0.020
*OSTC**	8.062	13.210	8.503	0.644	0.021
ZNF396	1.945	6.339	3.609	0.569	0.021
*HSPH1**	126.740	377.256	209.349	0.555	0.021
*WNK4**	3.435	15.104	7.881	0.522	0.022
*HSP90AA1**	1418.649	2332.492	1534.340	0.658	0.023
*PCDH17**	1.605	3.422	1.481	0.433	0.024
*RGS2**	9.686	34.538	18.252	0.528	0.025
*ZEB2**	1.240	3.439	1.540	0.448	0.028
*NPR2**	0.866	4.142	1.915	0.462	0.030
*CBLB**	3.734	7.831	4.258	0.544	0.030
*DNAJB4**	19.106	31.184	19.981	0.641	0.031
LOC100653021	2.687	4.252	2.012	0.473	0.031
*HSPA6**	0.234	80.982	42.000	0.519	0.032
*HSPA1A**	1787.859	5365.809	2561.155	0.477	0.033
*RC3H1**	3.065	6.591	3.711	0.563	0.034
IFITM10	1.406	3.702	1.660	0.448	0.034
C19orf77	0.212	3.349	0.638	0.191	0.036
*HSPA1B**	1774.210	5126.602	2489.364	0.486	0.039
*PCDH10**	0.829	3.141	1.418	0.451	0.040
*ORAI3**	2.925	4.221	2.083	0.493	0.042
*CYP39A1**	2.902	5.599	3.216	0.574	0.045
PCDH20	2.561	4.454	2.769	0.622	0.046
*LRRTM2**	0.585	5.903	1.437	0.243	0.047
*PYGM**	0.242	6.068	2.099	0.346	0.049
*DNAJC12**	2.395	3.744	1.684	0.450	0.050
**Up Regulated in Mutant *FOXE3***					
*GADD45A**	*18.679*	*23.046*	*39.312*	*1.706*	*0.049*
*CCND1**	*18.265*	*13.481*	*23.319*	*1.730*	*0.032*
*MAFK**	*8.342*	*8.361*	*14.997*	*1.794*	*0.023*
C2orf48	*3.548*	*5.014*	*9.878*	*1.970*	*0.015*
*HIST1H2AC**	*2.772*	*5.487*	*9.419*	*1.717*	*0.040*
*SLFN5**	*2.914*	*3.850*	*7.770*	*2.018*	*0.034*
*FBXW7**	*3.596*	*2.744*	*7.662*	*2.792*	*0.037*
*HIST1H2AG**	*1.368*	*2.392*	*5.017*	*2.097*	*0.034*

Supplementary Table 1: List of differentially expressed transcripts identified in cells expressing the mutant *FOXE3* using DNASTAR software. Asterisk denotes the transcripts expressed in mouse lens transcriptome.

Gene	Fold Change	Fold Change (log2)	P-value	Adjusted P-value
**Down regulated in Mutant *FOXE3***				
*COL3A1**	0.0662	-3.9181	1.6E-11	7.2E-10
*HSPA4L**	0.1120	-3.1590	6.3E-11	2.4E-09
C1ORF81	0.1469	-2.7670	4.2E-05	4.6E-04
*C16ORF90**	0.1986	-2.3318	1.7E-04	1.6E-03
KRTAP21-2	0.2155	-2.2144	3.1E-06	5.7E-05
CD5	0.2297	-2.1223	8.0E-04	5.0E-03
EFNA2	0.2464	-2.0209	1.6E-07	3.6E-62
*AEBP1**	0.2560	-1.9660	1.3E-04	1.3E-03
DUSP10	0.2610	-1.9377	2.5E-04	1.9E-03
AK074469	0.2998	-1.7378	4.6E-03	2.0E-02
*CNOT3**	0.3303	-1.5979	5.3E-06	7.6E-05
*PYGM**	0.3408	-1.5530	2.5E-05	3.0E-04
*ZNF398**	0.3422	-1.5470	4.2E-07	8.7E-06
*DNAJB1**	0.3509	-1.5107	1.7E-13	1.3E-11
*YWHAH**	0.3934	-1.3460	3.0E-04	2.2E-03
SLC25A34	0.3944	-1.3424	7.6E-10	2.5E-08
AZU1	0.4011	-1.3180	2.1E-04	1.8E-03
JSRP1	0.4068	-1.2977	4.2E-05	4.6E-04
LOC100129931	0.4072	-1.2961	1.4E-07	3.4E-06
F2	0.4171	-1.2615	4.8E-03	2.1E-02
MUC6	0.4717	-1.0839	5.9E-04	4.0E-03
*HSPH1**	0.4778	-1.0654	6.7E-04	4.3E-03
TMPRSS9	0.5333	-0.9070	4.5E-04	3.2E-03
*HSPE1**	0.5420	-0.8836	9.6E-03	3.5E-02
*MRPL18**	0.5510	-0.8600	3.5E-03	1.7E-02
SLC5A5	0.5608	-0.8344	1.2E-02	4.3E-02
GSDMD	0.5816	-0.7819	6.0E-03	2.5E-02
ZBTB18	0.5959	-0.7468	1.1E-03	6.5E-03
*FKBP4**	0.5984	-0.7408	4.1E-06	6.7E-05
*HSPA6**	0.6840	-0.5480	2.5E-05	3.0E-04
*RGS2**	0.7475	-0.4198	3.1E-03	1.5E-02
**Up regulated in mutant *FOXE3***				
*PIGV**	2.3810	1.2516	3.1E-19	3.6E-17
*COPZ2**	2.3062	1.2055	2.1E-05	2.7E-04
*DUSP8**	2.1008	1.0709	4.6E-06	7.0E-05
*ID3**	2.0803	1.0568	2.1E-12	1.2E-10
*TSC22D3**	1.9041	0.9291	2.1E-04	1.8E-03
*HIST2H2AA4**	1.7924	0.8419	1.2E-08	3.4E-07
*DPP7**	1.6663	0.7366	2.9E-04	2.2E-03
*HNRNPC**	1.6530	0.7251	5.4E-05	5.6E-04
*SMAD6**	1.5290	0.6126	1.9E-03	9.8E-03
HIST2H4A	1.5256	0.6094	6.2E-04	4.1E-03

Supplementary Table 2: **List of differentially expressed transcripts identified in cells expressing the mutant *FOXE3* using TopHat/DESeq algorithms.** Asterisk denotes the transcripts expressed in mouse lens transcriptome.
